# Sequence count data are poorly fit by the negative binomial distribution

**DOI:** 10.1371/journal.pone.0224909

**Published:** 2020-04-30

**Authors:** Stijn Hawinkel, J. C. W. Rayner, Luc Bijnens, Olivier Thas

**Affiliations:** 1 Department of Data Analysis and Mathematical Modelling, Ghent University, Ghent, Belgium; 2 Centre for Computer-Assisted Research in Mathematics and its Applications, School of Mathematical and Physical Sciences, University of Newcastle, Newcastle, Australia; 3 Quantitative Sciences, Janssen Pharmaceutical companies of Johnson and Johnson, Ghent, Belgium; 4 I-BioStat, Hasselt University, Hasselt, Belgium; 5 National Institute for Applied Statistics Research Australia (NIASRA), University of Wollongong, Wollongong, Australia; National Institute of Plant Genome Research (NIPGR), INDIA

## Abstract

Sequence count data are commonly modelled using the negative binomial (NB) distribution. Several empirical studies, however, have demonstrated that methods based on the NB-assumption do not always succeed in controlling the false discovery rate (FDR) at its nominal level. In this paper, we propose a dedicated statistical goodness of fit test for the NB distribution in regression models and demonstrate that the NB-assumption is violated in many publicly available RNA-Seq and 16S rRNA microbiome datasets. The zero-inflated NB distribution was not found to give a substantially better fit. We also show that the NB-based tests perform worse on the features for which the NB-assumption was violated than on the features for which no significant deviation was detected. This gives an explanation for the poor behaviour of NB-based tests in many published evaluation studies. We conclude that nonparametric tests should be preferred over parametric methods.

## Introduction

**Fig 1:**
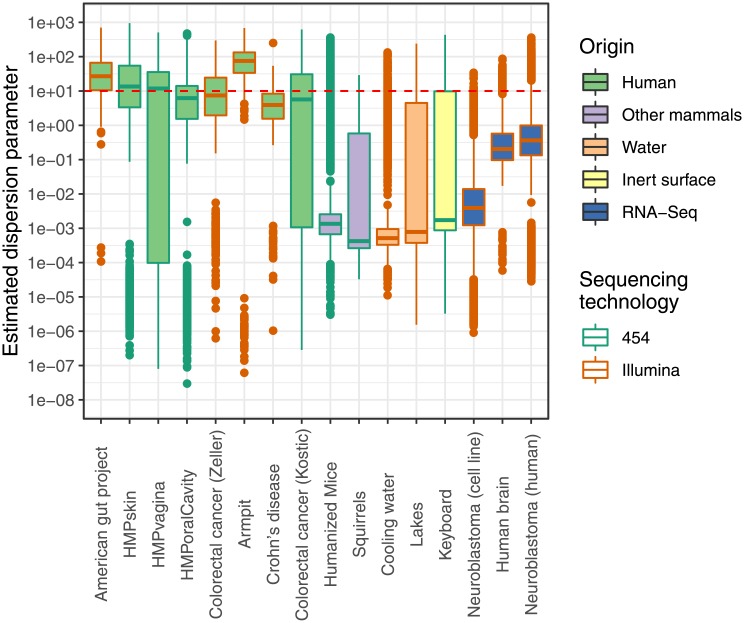
Boxplots of estimated feature-specific dispersion parameters of the negative binomial distribution per dataset. The red dashed line indicates the threshold above which the lack of fit to the negative binomial distribution could not be assessed reliably. See S4 Appendix for details on the datasets used.

In research areas such as RNA-sequencing (RNA-Seq) and microbiomics, sequencing technologies are applied to measure the composition of mixtures of nucleic acids [1, 2]. The resulting collection of sequences is then considered as a proxy for the transcriptomic state of a tissue or cell (in RNA-Seq) or for the species composition (for the microbiome). As both research areas employ the same technologies, their data properties and analysis techniques are similar. Apart from the biological variability between samples, the multiple manipulations, going from nucleic acid extraction, reverse transcription and PCR amplification to actual sequencing, introduce additional variability into the feature count tables. It is often assumed that the sequence counts from a single feature (either a taxon or a gene) follow the *negative binomial* (NB) distribution [3, 4]. The NB distribution can be seen as a extension of the Poisson distribution that allows for overdispersion due to the biological variability. This overdispersion is also strongly related to the frequency of zeroes in the count data. As evident from Fig 1, the overdispersion varies between features and depends on the biological nature of the samples, being notably large for microbiome data of human origin.

NB regression models [5] correct for sample-specific covariates such as *sequencing depth* or *library size*. Methods based on the NB distribution have been used for many purposes, such as clustering [6], discriminant analysis [7] and hypothesis testing [3, 8, 9]. In this paper we focus on the latter: testing for differential expression (RNA-Seq) and testing for differential abundance (microbiome). Good statistical hypothesis tests should be able to control the probability of a type I error (false positive result) at the nominal significance level and they should have sufficient power for detecting interesting biological results. Since with sequencing experiments not a single hypothesis is to be tested, but hundreds to thousands of hypotheses are tested simultaneously, these desirable properties of statistical tests can be reformulated as follows. The testing procedure (1) should be able to control the false discovery rate (FDR) at the nominal level, and (2) it should have sufficient sensitivity (i.e. true positive rate) for detecting interesting biological results.

Popular statistical tests for sequencing experiments include edgeR [10] and DESeq2 [11], which both rely on the NB assumption. These methods have been evaluated in many studies, using synthetic data generated with the very same NB distribution [6–8, 12]. As a self-fulfilling prophecy, the methods are then found to perform well, i.e. they appear to control the FDR and have good sensitivity. On the other hand, when evaluating the methods using realistically simulated data (e.g. by resampling from real datasets), comparative studies have revealed a substandard performance [13–16]. In particular, they conclude that the NB-based methods show a poor FDR control; often the FDR is larger than the nominal level, resulting in too many false positive findings. Nonparametric methods, which do not rely on strong distributional assumptions, have been demonstrated to control the FDR with realistically simulated data, e.g. the Wilcoxon rank sum test and ALDEx2 [17].

To date no conclusive explanation has been found for this drop in performance. One obvious possible explanation is that real sequence count data are not well described by the NB distribution. The goodness of fit of ecological count data to the NB distribution has been formally tested before, but not with theoretically supported tests. These investigations only considered a limited number of datasets and they did not find a lack of fit to the NB distribution [18–20].

In this paper we propose a new statistical goodness of fit (GoF) test for the NB distribution in regression models that are commonly used for analysing RNA-Seq and microbiome studies. The performance of the new GoF test is evaluated in a simulation study. When applied to publicly available RNA-Seq and microbiome datasets, a lack of fit was discovered for many features in several datasets. Finally, the consequences of these violations to the NB assumption are investigated.

## Smooth tests for the negative binomial distribution in regression models

### Negative binomial regression models

NB regression models are described here for a single feature. Let (*Y*_*i*_, ***x***_*i*_), *i* = 1, …, *n*, denote the *n* sample observations of outcome *Y* and the *p*-dimensional regressor ***x***. The regressor vector may include dummy variables for the coding of factor variables, and it may include a constant if an intercept is to be included in the model. We consider NB regression models of the form

$$Y|x∼\text{NB}(\mu \left(x_{i};\beta \right),\phi )$$

where *ϕ* is the overdispersion parameter and

$$\mu \left(x;\beta \right)=\text{exp}(\beta_{0}+\beta^{t}x)$$ (1)

in which *β*_0_ is a given constant, which is generally referred to as the offset of the regression model.

The smooth test will be constructed for the model used for comparing two experimental conditions (e.g. two treatments). This model includes an offset *β*_0_ = log(library size), an intercept and only one regressor, *x*_*i*_, defined as a 0/1 dummy variable referring to the two experimental conditions. Hence, $x_{i}^{t}=\left(1,x_{i}\right)$. Model 1 thus reduces to

$$\mu \left(x;\beta_{1},\beta_{2}\right)=\text{exp}(\beta_{0}+\beta_{1}+\beta_{2}x).$$ (2)

We further assume that, given the regressor *x*_*i*_, the outcomes *Y*_*i*_ are independently distributed.

### Construction of the test statistic

For the one-sample problem (i.e. testing the null hypothesis that a sample comes from a hypothesised distribution) many types of GoF tests have been proposed; see [21] for a comprehensive overview. Our test fits within the framework of smooth tests. For the one-sample problem this class of tests was first introduced by [22] and later generalised [23]. The main idea of smooth tests is to first embed the hypothesised distribution into a larger distribution that contains extra parameters, which we refer to as embedding parameters, such that when these extra parameters equal zero, the embedding distribution collapses to the hypothesised distribution. The GoF null hypothesis is thus equivalent to setting all embedding parameters to zero. The smooth test is then the efficient score test for this testing problem.

The extension of smooth tests from the one-sample problem to a regression setting, was first described in a PhD thesis [24]. Detailed theory and a few examples (normal, Poisson and zero-inflated Poisson regression) were recently provided [25]. In this section we give a brief outline of the construction. More details can be found in S1 Appendix.

Let *f*(*y*;*μ*, *ϕ*) denote the probability mass function of the negative binomial distribution with mean *μ* given by Eq 2. Thus *μ* depends on *x* and on *β*. In particular,

$$f\left(y;\mu ,\phi \right)=\text{P}\{Y=y\}=\frac{\Gamma (y+\phi^{-1})}{y!\Gamma (\phi^{-1})}{\left(\frac{\mu \phi }{1+\mu \phi }\right)}^{y}{\left(\frac{1}{1+\mu \phi }\right)}^{1/\phi },$$ (3)

where Γ(⋅) is the gamma function. Next, this distribution is embedded in a family of smooth alternatives,

$$f_{J}\left(y;\mu ,\phi ,\theta \right)=C\left(\mu ,\phi ,\theta \right)\text{exp}(\sum_{k=1}^{J}\theta_{j}h_{j}\left(y;\mu ,\phi \right))f\left(y;\mu ,\phi \right),$$ (4)

where *J* is the order of the smooth alternative, ***θ***^*t*^ = (*θ*^1^, …, *θ*_*J*_) is the vector of embedding parameters, *C*(*μ*, *ϕ*, ***θ***) is the normalisation constant, and {*h*_*k*_(*y*;*μ*, *ϕ*)} is a set of functions that are orthonormal on the hypothesised NB model. In particular, these functions must satisfy

$$\sum_{y=0}^{\infty }h_{j}\left(y;\mu ,\phi \right)h_{l}\left(y;\mu ,,\phi \right)f\left(y;\mu ,\phi \right)=\delta_{jl},$$ (5)

with *δ*_*jl*_ = 0 if *j* ≠ *l* and *δ*_*jl*_ = 1 if *j* = *l*.

The smooth test is basically the efficient score test for testing the null hypothesis *H*_0_: ***θ*** = **0** within the smooth family. Since the smooth family does not only contain the embedding *θ* parameters, but also the parameters *β* and *ϕ*, these nuisance parameters need to be estimated from the data and the score test needs to account for their estimation. We consider the method of maximum likelihood (ML) for parameter estimation. Note that this estimation method differs from the methods used for edgeR [10] and DESeq2 [11]. However, our choice does not undermine the credibility of the results of our testing procedure: as long as the smooth test controls the type I error and has power, it is a valid testing procedure. Changing the estimation procedure, as part of the data analysis pipeline, does not alter the validity of a distributional assumption.

The next few paragraphs describe the ML estimation procedure, and shows the construction of the efficient score test statistic. For a score test we only need the ML estimators (MLE) of *β* and *ϕ* under the null hypothesis, i.e. in the hypothesised NB regression model given by probability mass function of Eq 3. First the log-likelihood function is constructed,

$$l\left(\beta ,\phi \right)=\sum_{i=1}^{n}\text{log}\;f\left(y_{i};\beta ,\phi \right).$$

Next, the score statistics for the parameters *β* and *ϕ* are computed,

$$\begin{array}{ccc} \frac{\partial }{\partial \beta }l\left(\beta ,\phi \right) & = & \sum_{i=1}^{n}\frac{\partial }{\partial \beta }\text{log}\;f\left(y_{i};\beta ,\phi \right)=\sum_{i=1}^{n}\frac{x_{i}\left(y_{i}-\mu_{i}\right)}{1+\mu_{i}\phi }\\ \frac{\partial }{\partial \phi }l\left(\beta ,\phi \right) & = & \sum_{i=1}^{n}\frac{\partial }{\partial \phi }\text{log}\;f\left(y_{i};\beta ,\phi \right)\\ & = & \sum_{i=1}^{n}(\Psi \left(y_{i}+\phi^{-1}\right)-\Psi \left(\phi^{-1}\right)-\text{log}\;\left(1+\phi \mu_{i}\right)+1-\frac{y_{i}\phi +1}{1+\phi \mu_{i}}), \end{array}$$

with Ψ(⋅) the digamma function. We will use the notation

$$\begin{array}{cc} & s_{\beta }\left(y_{i};\beta ,\phi \right)=\frac{\partial }{\partial \beta }\text{log}\;\;f\left(y_{i};\beta ,\phi \right)\\ & s_{\phi }\left(y_{i};\beta ,\phi \right)=\frac{\partial }{\partial \phi }\text{log}\;f\left(y_{i};\beta ,\phi \right) \end{array}$$ (6)

for the score functions of *β* and *ϕ*, respectively.

The MLEs of *β* and *ϕ*, subsequently denoted by $\hat{\beta }$ and $\hat{\phi }$, are the solution to the system of equations $\frac{\partial }{\partial \beta }l\left(\beta ,\phi \right)=0$ and $\frac{\partial }{\partial \phi }l\left(\beta ,\phi \right)=0$. No analytical solution is available, and so an iterative numerical algorithm is needed.

In the presence of nuisance parameters, there generally are two approaches for the construction of efficient score tests. Either the information matrix is corrected to account for the estimation, or the orthonormal functions in the smooth family are altered to make them also orthogonal to the score functions of the nuisance parameters. We take the latter route. In particular, we require in addition to the orthonormality condition of Eq 5 that the *h*-functions also satisfy

$$\sum_{y=0}^{\infty }h_{j}\left(y;\mu ,\phi \right)s_{\beta }\left(y;\mu ,\phi \right)f\left(y;\mu ,\phi \right)=\sum_{y=0}^{\infty }h_{j}\left(y;\mu ,\phi \right)s_{\phi }\left(y;\mu ,\phi \right)f\left(y;\mu ,\phi \right)=0$$ (7)

for all parameter values. For the implementation of the test in this paper, we have opted for polynomial *h*-functions because the resulting score test statistics have an interpretation related to deviations from the hypothesised NB distribution in terms of moments [21, 23, 26]. For example, the score test statistic based on the third order polynomial, say *h*_3_, can detect deviations from the NB distribution in terms of the third order moment (skewness). Similarly, the statistic based on the fourth order polynomial, say *h*_4_, detects deviations in the fourth order moment (kurtosis). In S1 Appendix it is shown how the Gram-Schmidt orthogonalisation procedure can be used for constructing the orthonormal polynomials. Note that the orthonormal constraints, expressed by Eqs 5 and 7, should hold for all *μ* and all *ϕ*. Since the former parameter equals exp(*β*_0_ + *β*_1_ + *β*_2_*x*), the orthonormal polynomials should be computed separately for the two experimental condition groups (*x* = 0 and *x* = 1), and in practice the unknown parameters should be replaced by their MLEs.

For notational comfort we denote the vector of nuisance parameters (here: ***β*** and *ϕ*) by $\eta^{t}=\left(\beta ,\phi \right)$. The smooth test statistic is a score test statistic which requires the score functions for the *θ* parameters in the smooth family of alternatives (Eq 4), evaluated under the null hypothesis. For parameter *θ*_*k*_, this restricted score function is given by

$${s_{\theta ,k}\left(y;x,\eta ,\theta \right)|}_{\theta =0}={\frac{\partial }{\partial \theta_{k}}\text{log}\;f_{J}\left(y;x,\eta ,\theta \right)|}_{\theta =0}=h_{k}\left(y;x,\eta \right).$$

Define

$$\begin{array}{c} V_{k}\left(\eta \right)=n^{-1/2}\sum_{i=1}^{n}h_{k}\left(y_{i};x_{i},\beta \right) \end{array}$$

and ***V***^*t*^ = (*V*_1_(***η***), …, *V*_J_(***η***)).

The score test statistic is given by

$$T_{J}=V{\left(\hat{\eta }\right)}^{t}{\left(I^{-1}\left(\hat{\eta }\right)\right)}_{\theta \theta }V\left(\hat{\eta }\right),$$ (8)

where ***I***(***η***) is the Fisher information matrix for the parameter vector (***η***, ***θ***) in the smooth alternative, evaluated under the null hypothesis. In S1 Appendix we demonstrate that this matrix equals *n* times the identity matrix. This simple form is a consequence of the orthonormality conditions imposed on the orthonormal *h*-functions. The expression ${\left(I^{-1}\left(\hat{\eta }\right)\right)}_{\theta \theta }$ in Eq 8 refers to the elements in the inverse Fisher information matrix that refers to the *J*-dimensional ***θ*** vector, and hence it equals *n*^−1^ times the identity matrix. Thanks to this diagonal structure, we can write the test statistic *T*_*J*_ as a decomposition of *J* components, i.e.

$$T_{j}=V_{1}^{2}\left(\hat{\eta }\right)+\cdots +V_{J}^{2}\left(\hat{\eta }\right).$$

The distribution theory provided in [23] and [25] is directly applicable, resulting in the asymptotic null distributions of *T*_*J*_ and $V(\hat{\eta })$. Under the null hypothesis, as *n* → ∞,

$$V(\hat{\eta })\overset{d}{⟶}\text{MVN}(0,I)\;T_{J}\overset{d}{⟶}\chi_{J}^{2}.$$

Since this is an asymptotic result, its practical validity for realistic sample sizes should be empirically investigated in a simulation study. This is the topic of the next section. In particular, the parametric bootstrap will be assessed. The parametric bootstrap constructs the null distribution of the test statistic based on repeatedly sampling *n* sample observations from the fitted hypothesised model. For the one-sample problem, the parametric bootstrap is described in detail in [23], but this procedure needs to be adjusted to the regression setting. Details are given in S2 Appendix.

### Simulation study

According the theory of [25], the smooth test statistic *T*_*J*_ asymptotically has a $\chi_{J}^{2}$ null distribution and the components $S_{k}\left(\hat{\eta }\right)$ asymptotically have standard normal distributions. Still, this does not guarantee that the use of these limiting distributions in practical settings with rather small sample sizes, gives good type I error control. In this section we empirically evaluate the null distribution of the test statistic and its *p*-value for realistic sample sizes. We also evaluate the tests with *p*-values calculated based on the parametric bootstrap procedure. For most smooth tests the bootstrap procedure gives good results [23].

We produced 6 datasets, each with counts randomly generated from NB distributions with increasing overdispersion parameter (*ϕ* ∈ {0.01, 0.1, 1, 10, 20, 30}). Each dataset has *p* = 500 features in 50 samples balanced over two treatment groups. The mean of the NB distribution for feature *j* in sample *i* is given by log*μ*_*ij*_ = *β*_0*i*_ + *β*_1*j*_ + *β*_2_*x*_*i*_, where *x*_*i*_ is a 0/1 dummy coding for the treatment group, the offsets *β*_0*i*_ (log-library sizes) are sampled from a normal distribution with mean 9.21 and standard deviation 1.15. The feature baselines *β*_1*j*_ are sampled from a uniform distribution over the interval [-13.8, -6.91], and the log-fold change *β*_2_ is fixed to 2. These settings correspond to the parameter values observed in real datasets.

**Fig 2:**
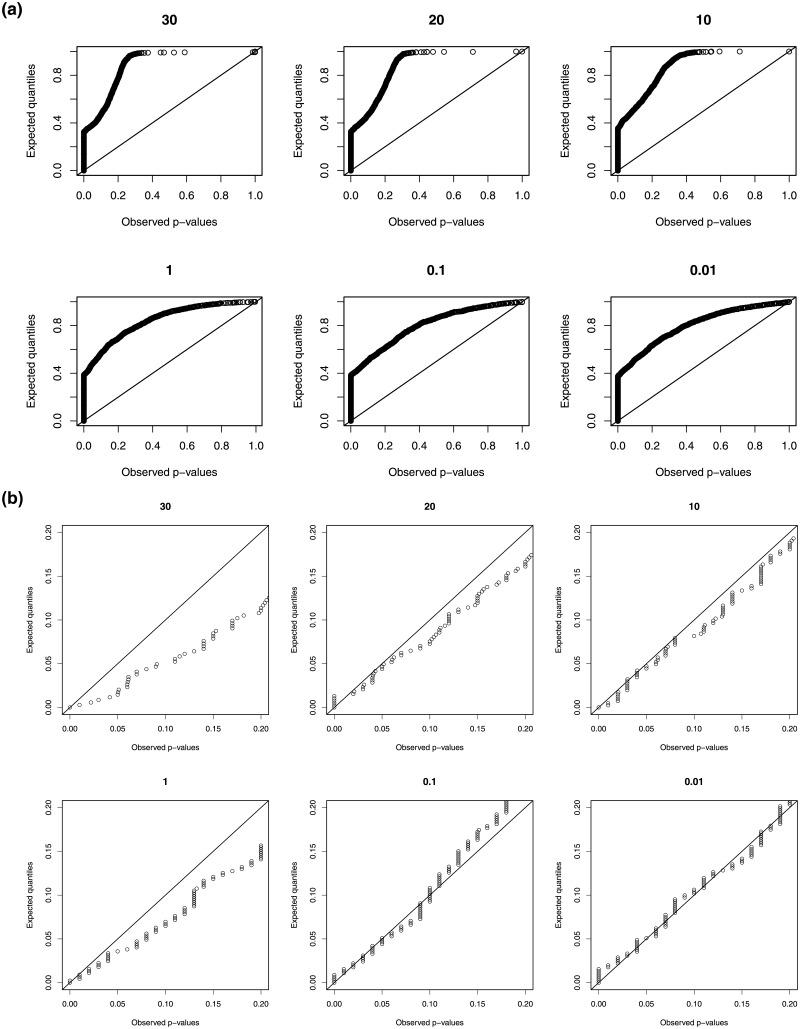
The uniform QQ plots of 500 p-values based on (a) the asymptotic null distribution and on (b) the bootstrap. The overdispersion parameter is printed on top of each panel.

The results are shown in Fig 2. All QQ-plots of p-values from the asymptotic *χ*^2^ approximation show a substantial deviation from the uniform distribution. This indicates that for a sample size of 50 this asymptotic approximation cannot be reliably used as it will not result in a control of the type I error rate. The QQ-plots for the bootstrap p-values, on the other hand, show good resemblance to the uniform distribution, unless the overdispersion parameter is larger than 10. Moreover, the resemblance to the uniform distribution is particularly close for small p-values, which is important for the type I error rate control.

We may conclude that the parametric bootstrap is a reliable method for p-value calculation for the tests developed in this paper, unless the overdispersion is larger than 10. The good approximation to the uniform distribution is also necessary for good FDR control in the multiple testing context. In S3 Appendix also the sampling distribution of the estimator of the overdispersion parameter is investigated. For extreme small or large values of the overdispersion parameter, bias and non-normality of the estimator is observed. This may be part of the explanation of the poor behaviour of the goodness of fit test for such extreme overdispersions.

### Estimating the proportion of features with lack-of-fit

In this section, the goal is to estimate the number of features poorly fit by the NB distribution, rather than identifying which features show a deviating distribution. For this we leverage the fact that many features are being tested simultaneously, using the method developed by [27]. It relies on the property that the distribution of the p-values is a mixture distribution of p-values of features for which the null hypothesis holds, and p-values of features for which the null hypothesis does not hold. The density function of the p-values can then be described as

$$g\left(p\right)=\pi_{0}g_{0}\left(p\right)+\left(1-\pi_{0}\right)g_{A}\left(p\right),$$

where *g*_0_(*p*) is the density of p-values under the null distribution, *g*_*A*_(*p*) the density of p-values under the alternative distribution and *π*_0_ the mixing proportion. The distribution *g*_0_(*p*) is known to be uniform on [0, 1], and the distribution *g*_*A*_(*p*) is skewed to the right. If we assume that there is some *p*_*c*_ for which *g*_*A*_(*p*|*p* > *p*_*c*_) = 0, we may employ the fdrtool R-package [28] for the estimation of *p*_*c*_. [27] proposed a modified Grenander density estimator for *g*(*p*). This estimator is similar to a nonparametric estimator of *g*(*p*), with the additional restriction of being monotonically decreasing (since *g*_*A*_(*p*) is monotonically decreasing with *p*, and *g*_0_(*p*) is uniform, *g*(*p*) must also be monotonically decreasing). The mixing proportion *π*_0_ is then estimated as

$${\hat{\pi }}_{0}=\text{min}(1,\frac{\sum_{k=1}^{p}I\left(p_{k}>p_{c}\right)}{p}\frac{1}{1-\hat{G}\left(p\right)})$$

with $\hat{G}\left(p\right)$ the distribution function corresponding to the estimate $\hat{g}\left(p\right)$. The quantity we are interested in is 1 − *π*_0_, i.e. the proportion of features that is not well fit by the NB. This approach has a very particular advantage: there is no need for very precise p-values for this estimation procedure. This means that a small number of bootstrap samples suffice, which greatly reduces the computational burden.

Finally, we note that even when a comparatively small percentage of features does not follow the NB distribution, this may still affect the analysis of an entire dataset. Typically, the negative binomial regression model is used to perform one test per feature. Next, one implements a multiple testing correction that works on the ensemble of p-values. Hence, a violation of the assumptions for some features can also affect the inference for features that *do* follow the negative binomial distribution.

## Application to sequencing data

**Fig 3:**
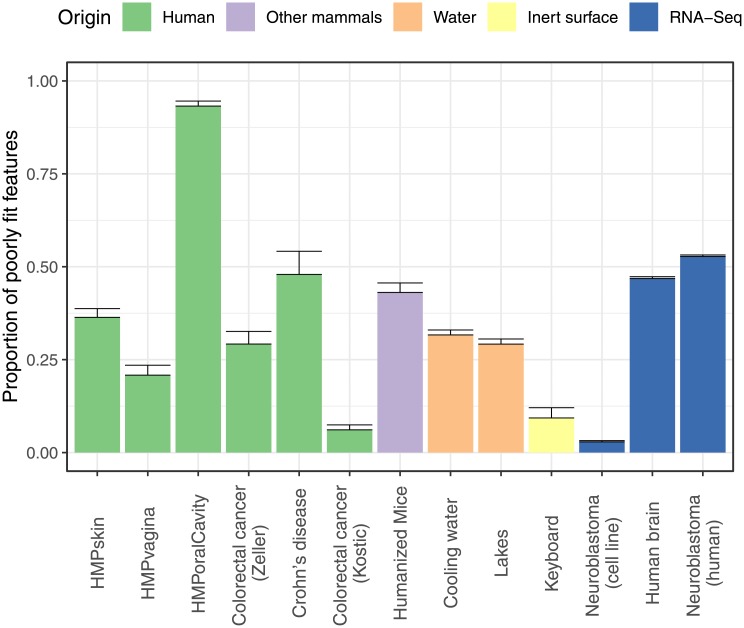
Estimated proportions of features with lack of fit to the negative binomial distribution per dataset. Error bars represent standard errors.

The new smooth test with *J* = 4 was applied to a wide range of datasets: microbiome data from human [29–34], animal [35, 36], inert surface [37] and freshwater [38, 39] origin, as well as RNA-seq data [14, 40, 41]. More details on these datasets can be found in S4 Appendix. Features with an estimated overdispersion parameter of 10 or larger were omitted from the analysis so as to guarantee the validity of the bootstrap testing procedure (See Fig 1 for the distribution of the estimates of the overdispersion parameters). For the other features, the bootstrap (with 1,000 bootstrap runs) was used for *p*-value calculation. Subsequently, the method of the previous section was applied for estimating, for each dataset, the fraction of features poorly fit by the NB distribution. The results are summarised in Fig 3. The fraction of non-NB distributed features ranges from 2% to 90%, with many datasets having more than 25% of the features deviating from the NB assumption. For completeness we have repeated the analysis using all features; see S1 Fig. Although not all bootstrap p-values can be trusted, this analysis gives the same overall conclusion.

**Fig 4:**
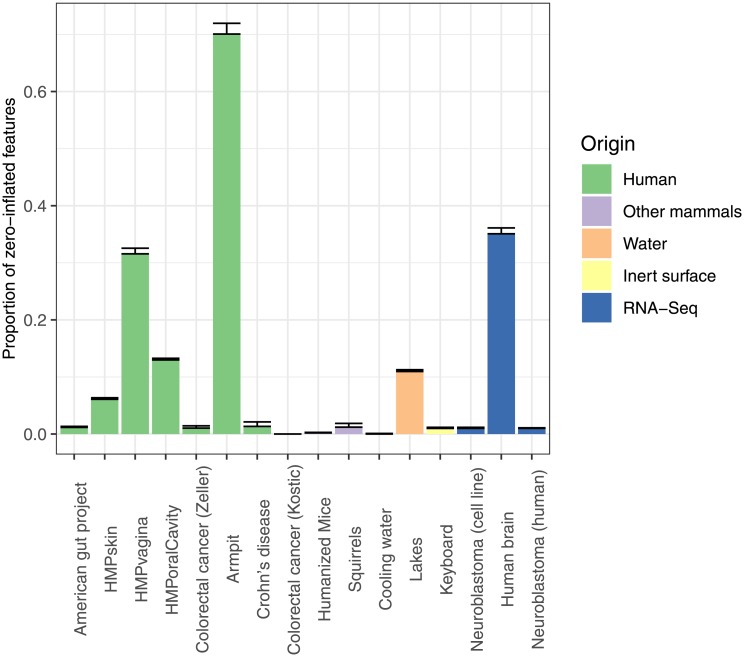
Estimated proportion of features with significant zero-inflation with respect to the NB distribution. Error bars represent standard errors.

Zero-inflated models have been proposed as an improvement of the NB distribution [42–45]. All features were tested with a likelihood ratio (LR) test for comparing the NB against a zero-inflated negative binomial (ZINB) distribution. Based on these p-values, the fraction of significant features was estimated as before. Some exceptions notwithstanding, most of the datasets did not exhibit zero-inflation with respect to the NB distribution, or not for sufficiently many features to explain the observed lack of fit to the NB distribution (see Fig 4). Note that this LR test only serves to compare the NB and ZINB models, and does not provide evidence that the ZINB distribution as such fits the data well. It is also important to remark that these proportions only refer to the features for which a ZINB model could be fitted and may therefore overestimate the real proportion of zero-inflated features (S1 Table shows the proportions of features without zeroes and for which hence no ZINB distribution can be fitted. Note that particularly the RNA-Seq datasets show many features without zero counts).

In S5 Appendix we further investigated possible data-related features that could explain the lack of fit to the NB assumption, but no very clear conclusions can be formulated. We observe that poorly fit features are often among the high abundant features with intermediate zero frequencies. Moreover, the fit to the NB is worse in samples with large library sizes.

## Consequences

From the results from the previous section it is clear that many features in sequence count data do not follow the NB distribution. It has been shown before that the performance of statistical tests that rely on the NB distribution deteriorates when applied to realistically simulated data [13–16]. In this section we investigate the validity of NB-based hypothesis tests when applied to features that exhibited a lack of fit to the NB distribution.

Based on the bootstrap p-values of the smooth test, q-values are obtained from the procedure of [27]. All features with q-values smaller than 10% are grouped together and considered to be “poorly fit features”. The other features are considered to be well fit by the NB distribution.

**Fig 5:**
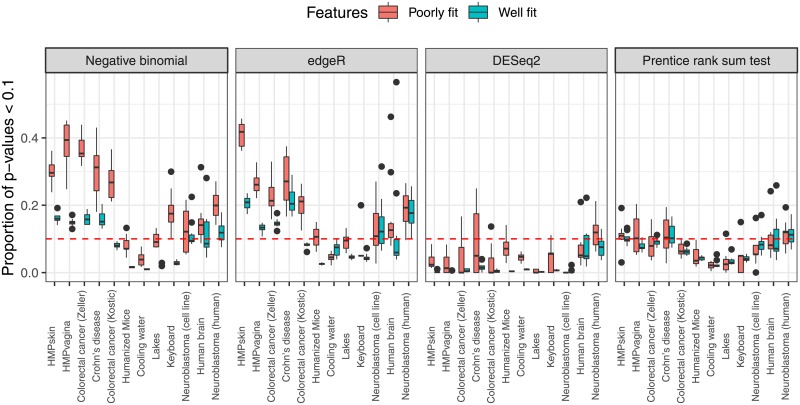
Boxplots of proportions of p-values smaller than 0.1 for features with poor and good fit to the NB distribution, per dataset. The p-values are computed with the Wald test in a NB model, edgeR, DESeq2 and with the Prentice rank sum test.

For all datasets the following simulation procedure is followed. Samples are randomly allocated to one of two groups with equal probability (similar to the “real data shuffling” in [13]). Next, to every feature a negative binomial regression model is fitted with maximum likelihood, with the library sizes as offset and the original grouping variable (S4 Appendix) and the random grouping variable as regressors. P-values for the Wald test for the random grouping variable are calculated for every feature. In a similar fashion edgeR and DESeq2 were applied, as well as the nonparametric Prentice rank sum test [46]. The latter test is an extension of the Wilcoxon rank sum and Friedman tests, and it can be used for testing the effect of the random grouping variable, while accounting for the additional grouping variable. All p-values are expected to be distributed as the [0, 1] uniform distribution. This procedure is repeated 50 times for each dataset. The HMP oral cavity dataset was omitted from this study, as all features had q-values below 0.1. Fig 5 shows the proportions of p-values smaller than 0.10 for both groups of features and for all datasets. Results are shown for the four tests for differential abundance/expression. If the proportion is close to 0.1, then the test is able to control the type I error at this level. The full uniform QQ-plots (S2 Fig) are more informative, but overall the conclusions are the same.

The results show that for the Wald and edgeR tests, which both rely on the NB-distribution, the poorly fit features generally show a larger proportion of small p-values than the features for which no lack of fit to the NB was detected. This demonstrates that the poor FDR control of NB-based tests can be due to the poor fit of the NB-distribution to real sequencing count data. Overall, DESeq2 gives much better results than the other two NB-based tests. We can also see a difference depending on the origin of the datasets. For the human microbiome datasets we see large proportions, corresponding to increased type I error rates. The same conclusion holds for the RNA-Seq datasets, but to a lesser extent. The results for the environmental microbiome datasets suggest conservative type I error rate control.

For the nonparametric Prentice rank sum test, no large difference between poorly and well fit features can be observed. Moreover, the proportions are not often larger than 0.10, indicating that this test controls the type I error. This can be expected, because the test does not rely on the NB distributional assumption.

## Conclusion and recommendation

Sequencing count data are often assumed to follow the NB or ZINB distributions, which form the basis of several statistical procedures for testing for differential expression (RNA-Seq) or differential abundance (microbiome). When such statistical methods are evaluated in parametric simulation studies, the count data are often generated from the same distributions. These statistical tests then seem to perform well, in the sense that they control the FDR and have good sensitivity. This is, of course, a self fulfilling prophecy. On the other hand, when these methods are evaluated in simulation studies with realistically simulated data, these NB-based methods show a poor FDR control. In particular, their true FDR is frequently much larger than the nominal level, leading to too many false positive results. The consequence of such poor FDR control, is that too many features (genes or taxa) will be called differentially expressed or abundant, potentially resulting in the publication of many false positive results, and hence contributing to the issue of poor reproducibility of scientific publications [13–16]. Nonparametric methods (the Wilcoxon rank sum test and ALDEx2), on the other hand, generally perform better in terms of controlling the FDR, but they have smaller sensitivity.

In an attempt to understand the cause of the poor FDR control of NB-based methods, we aimed to assess this distributional assumption in several public RNA-Seq and microbiome sequencing count datasets. For this purpose, we had to develop a new statistical goodness of fit test for the NB assumption in regression models, such that library size could be accounted for, and data from several experimental groups (e.g. treatments) could be simultaneously tested. This approach allows us to use more data for a single hypothesis test, hence increasing the power of the test. We developed a new smooth goodness of fit test, which was empirically evaluated in a simulation study, from which we conclude that the bootstrap version of the test gives valid results (i.e. have uniform p-value distribution under the null hypothesis) as long as the overdispersion of the NB distribution is not larger than 10.

We analysed 13 sequencing count datasets with the new goodness of fit test and we estimated the proportion of features that does not obey the NB assumption. We conclude that most datasets have more than 25% of their features deviating from the NB assumption, and 5 out of the 13 datasets have more than 40% of the features that cannot be described by the NB distribution. We also checked whether the ZINB distribution does significantly better fit to the data, but apart from 3 datasets, no considerably better fit was observed.

Finally, the features with lack of fit to the NB distributions were found to be more prone to incur false positive findings in statistical tests that rely on the NB distribution, than well fit features. On the other hand, the nonparametric rank sum test performed equally well for well fit and poorly fit features, and managed to control the type I error rate.

Researchers can thus apply our new goodness of fit test to their sequence count datasets, to see if the NB distribution provides a good fit to them. The result of this test should then guide the choice of hypothesis test, e.g. for differential abundance or expression detection. If the fit is poor, we recommend to use the nonparametric tests to analyse these data.

## Supporting information

S1 Fig Poorly fit features. Barplots of the estimated proportion of features poorly fit by the negative binomial distribution, when using all features (also those with dispersion >10). Error bars represent standard errors.
(EPS)

S2 Fig Uniform QQ-plots. Uniform QQ-plots of the *p*-values of four tests (Wald test in a NB model, edgeR, DESeq2 and with the Prentice rank sum test) for all datasets evaluated under Mock simulations (50 simulation runs). QQ-plots for poorly and well fitted features are shown.
(EPS)

S1 Appendix Construction of the test statistic. In this appendix more details are given about the construction of the smooth test statistic.
(PDF)

S2 Appendix The parametric bootstrap. In this appendix more details are given about parametric bootstrap procedure for p-value calculations.
(PDF)

S3 Appendix Estimation of the overdispersion parameter. In this appendix results of a simulation study are presented, aimed at investigating the sampling distribution of the MLE of the overdispersion parameter for a range of overdispersion values.
(PDF)

S4 Appendix Datasets. In this appendix details are provided about the datasets used in the paper.
(PDF)

S5 Appendix Exploration of the lack of fit. In this appendix the relationship between the fit to the NB distribution and some data-related features are graphically investigated.
(PDF)

S6 Appendix Datasets and R-code. All datasets used in the analysis, together with the R-code used to produce the outputs.
(GZ) Click here for additional data file.

S1 Table Zero observations. Proportions of features without zero observations in all datasets under study.
(PDF)
