# Supplementary material for: Sequence count data are poorly fit by the negative binomial distribution
Source: PLoS One. 2020 Apr 30;15(4):e0224909. doi: 10.1371/journal.pone.0224909 (PMC7192467; doi:10.1371/journal.pone.0224909)
Supplement: S1 Appendix — In this appendix more details are given about the construction of the smooth test statistic. (PDF) [file pone.0224909.s003.pdf]

S1 Appendix: Construction of the smooth test statistic

1 Introduction to smooth tests

Smooth goodness of fit tests were initially introduced by Neyman [1], and later extended by Rayner and Best [2]. We refer to the monograph of [3] for a comprehensive overview of smooth tests. These smooth tests were developed for testing the null hypothesis that a sample of i.i.d. observations comes from a hypothesized distribution, which may depend on a vector of nuisance parameters. This problem is known as the *one-sample problem*. In our context, however, the sample observations are not identically distributed: their means depend on an offset and on regressors. Rippon [4] further extended smooth tests to regression models, but the negative binomial regression model was not included. In this document we derive a smooth test for the NB regression model; the construction is slightly different from the traditional approach.

The null hypothesis of interest is that the n sample observations Y are distributed with the NB density function $f(y;\mu_i,\phi)$ for each of its elements i ($i=1,\ldots,n$), with mean μ_i and dispersion parameter ϕ. The parameters μ_i (or the β-parameters involved in the model for μ_i) and ϕ are not fixed under the null hypothesis, and hence should be estimated from the data. In the context of goodness of fit testing, these parameters are referred to as *nuisance parameters*.

The construction of a smooth test generally starts with embedding the hypothesized density function into a broader family of density functions. This broader family is known as the *smooth alternative*, because it varies smoothly from the hypothesized density function. In particular, this smooth alternative f_{sa} for observation i is of the form

$$f_{sa}(y;\mu_i,\phi,\boldsymbol{\theta}) = C(\boldsymbol{\theta},\mu_i,\phi)\exp\Big(\sum_{j=1}^{J}\theta_j h_j(y;\mu_i,\phi)\Big)f(y;\mu_i,\phi) \tag{1}$$

with $\{h_j(y;\mu_i,\phi)\}$ a set of basis functions (see further for more details), $\boldsymbol{\theta}^t=(\theta_1,\ldots,\theta_J)$ a vector of embedding parameters, and $C(\boldsymbol{\theta},\mu_i,\phi)$ is the normalization constant (to make the sum of the smooth alternative over $y=0,1,\ldots$ converge to 1). Note that if $\boldsymbol{\theta}=\mathbf{0}$ the smooth alternative collapses to the hypothesized density function. Hence, the original goodness of fit null hypothesis is now reduced to $\boldsymbol{\theta}=\mathbf{0}$. Since the θ-parameters appear in a proper density function, the hypothesis testing can be performed in a likelihood-framework. In particular, smooth tests are basically score tests, which have the practical advantage that parameters only need to be estimated under the null hypothesis (i.e. in the NB regression model) and hence the θ-parameters do not need to be estimated.

The choice of the basis functions $\{h_j(y; \mu_i, \phi)\}$ gives flexibility to the researcher to focus the power of the smooth test on alternatives of primary interest. Often such alternatives are not well known or such alternatives are formulated in general terms. For example, alternatives for which the variance, skewness or kurtosis are not in agreement with the hypothesized family of distributions. In that case, the basis functions are often chosen to be polynomial, i.e. h_j is a polynomial of degree j. We refer to [3] and [5] for detailed discussions on the interpretation of smooth tests in terms of deviating moments. If no nuisance parameters are to be estimated (e.g. the $\boldsymbol{\beta}$ and ϕ parameters in the NB distribution are known), a convenient choice of polynomials is given by a set of *orthonormal* polynomials on the hypothesized distribution, i.e. the polynomials $h_j(y; \mu_i, \phi)$ must satisfy

$$\sum_{y=0}^{+\infty} h_j(y; \mu_i, \phi) h_l(y; \mu_i, \phi) f(y; \mu_i, \phi) = \delta_{jl} \tag{2}$$

with $\delta_{jl} = 1$ if $j = l$ and 0 otherwise. With this choice, the score test statistics for testing $\theta_j = 0$ and $\theta_l = 0$ are asymptotically independently distributed under the null hypotheses, which results in a smooth test statistic (for jointly testing that all θs are zero) that can be conveniently written as a sum of such components. However, when nuisance parameters need to be estimated, the independence property often vanishes. This does not necessarily affect the power of the test, but the smooth test statistic no longer has a simple form as the sum of components. Independence can be regained by orthogonalising the basis functions to the score functions of the nuisance parameters. We call $\boldsymbol{\eta}^t = (\boldsymbol{\beta}^t, \phi) = (\eta_1, ..., \eta_{d+1})$ the vector of nuisance parameters of the negative binomial distribution, and $s_m(y; \eta_m) = \partial \log f(y; \mu_i, \phi)/\partial \eta_m$ the score function of the nuisance parameter η_m in the hypothesized NB model. We now also require for all $j = 1, \ldots, J$ and for all $m = 1, \ldots, d+1$,

$$\sum_{y=0}^{+\infty} h_j(y; \mu_i, \phi) s_m(y; \eta_m) f(y; \mu_i, \phi) = 0. \tag{3}$$

This orthogonalisation to the nuisance score functions is, however, not part of the conventional approach to the construction of smooth tests, but the resulting test is equivalent to the conventionally constructed smooth test. Basis functions satisfying (2) and (3) directly point in directions of the important alternative, without "spoiling" power in directions that are consistent with the hypothesized model.

For some distributions the score functions of the nuisance parameters coincide with one or more basis functions. This often happens for nuisance parameters related to the mean and variance, for which the score functions are polynomials of order one and two. In that case, the corresponding components of $\boldsymbol{\theta}$ are exactly zero.

In these circumstances the corresponding terms are first removed from the smooth alternative (1) before constructing the smooth test. For other distributions, the score functions of the nuisance parameters do not coincide with basis functions, but they are only approximately linearly dependent on the hypothesized distribution (i.e. their inner product as defined in (3) is large). This happens e.g. for nuisance parameters related to the mean and the variance of distributions for which maximum likelihood and method of moments estimators are different. The NB distribution is one example. It has been argued that for these distributions, the corresponding terms may also be removed from the smooth alternative [3].

The order J of the smooth alternative can be either fixed to a rather small order (4, 5, or 6) or an adaptive procedure can be used for choosing J based on the data (the maximal order J may then often be set to 10). The advantage of the latter is that the resulting test will have power for a larger family of alternatives, but the adaptive test looses some power for the lower order alternatives. Since we believe that particularly the lower order alternatives, which deviate from the NB model in e.g. skewness and kurtosis, are the more important, we will fix $J = 4$. We refer to [3] and [5] for comparisons between fixed J and data-driven J based tests.

2 Smooth tests for the negative binomial distribution

In this section the construction of the smooth test for the NB distribution of order $J = 4$ is given in some detail.

2.1 Basis functions

We start from a set of polynomial basis functions. In the smooth alternative (1) the terms related to the first two polynomial basis functions are removed, because these basis functions are very close to the nuisance parameter score functions.

2.1.1 Gram-Schmidt orthogonalization

We will use a more generic notation for the orthonormality conditions in (2) and (3). Orthogonalisation can be seen as the calculation of residuals of elements in a Hilbert space, after orthogonal projection onto another element. Smooth tests are constructed in the Hilbert space of functions of $y \in \mathbb{N}$, equipped with inner product given by

$$\langle a, b \rangle_f = \sum_{y=0}^{+\infty} a(y)b(y)f(y), \tag{4}$$

for arbitrary Hilbert space elements a and b and for a density function f. Note that this inner product, and hence the Hilbert space, is defined by density function f. If f is the NB density $f(y; \mu_i, \phi)$, this means that for each pair of μ_i (or, equivalently, $\boldsymbol{\beta}$) and ϕ, another Hilbert space is defined. In what follows, the dependence on f will often be omitted; so we will simply write $\langle a, b \rangle$.

As before, with $\boldsymbol{\eta}^t = (\boldsymbol{\beta}^t, \phi)$ the vector of nuisance parameters, the score function of $\boldsymbol{\eta}$ is denoted by $s(y; \boldsymbol{\eta}) = \partial \log f(y; \mu_i, \phi)/\partial \boldsymbol{\eta}$. For simplicity, we assume that there is only one regressor (d=2) such that $\boldsymbol{\beta}^t = (\beta_0, \beta_1)$ and $\mathbf{x}_i^t = (1, x_{i1})$.

Gram-Schmidt orthogonalization is a procedure to produce a function that is orthogonal to a given set of functions. We use it to construct the orthogonal basis functions h_j orthogonal to the nuisance score function s and all previous basis functions $h_0, h_3, \ldots, h_{j-1}$. Note that h_1 and h_2 are removed, because they are too close to the nuisance score functions. The (normalised) polynomial of order zero is $h_0(y) = 1$.

The procedure is initialised with an arbitrary polynomial of order 3. We take the simple form,

$$h_3^{\text{init}}(y) = 1 + y + y^2 + y^3$$

and apply the Gram-Schmidt orthogonalization procedure:

$$h_3(\cdot; \boldsymbol{\eta}) = h_3^{\text{init}}(\cdot) - \langle h_3^{\text{init}}, s_{\beta_0}(\cdot; \boldsymbol{\eta}) \rangle \frac{s_{\beta_0}(\cdot; \boldsymbol{\eta})}{\|s_{\beta_0}(\cdot; \boldsymbol{\eta})\|} - \langle h_3^{\text{init}}, s_{\beta_1}(\cdot; \boldsymbol{\eta}) \rangle \frac{s_{\beta_1}(\cdot; \boldsymbol{\eta})}{\|s_{\beta_1}(\cdot; \boldsymbol{\eta})\|} - \langle h_3^{\text{init}}, s_{\phi}(\cdot; \boldsymbol{\eta}) \rangle \frac{s_{\phi}(\cdot; \boldsymbol{\eta})}{\|s_{\phi}(\cdot; \boldsymbol{\eta})\|} - \langle h_3^{\text{init}}, h_0 \rangle \frac{h_0}{\|h_0\|}$$

in which s_{β_0}, s_{β_1} and s_ϕ are the components of score function $s(y; \boldsymbol{\eta})$ and $\| \cdot \|$ denotes the squared norm (the inner product (4) of a Hilbert space element with itself). With this h_3, the function h_4 is computed by applying the Gram-Schmidt procedure, starting from

$$h_4^{\text{init}}(y) = 1 + y + y^2 + y^3 + y^4.$$

In particular,

$$h_4(\cdot;\boldsymbol{\eta}) \;=\; h_4^{\text{init}}(\cdot) - \left\langle h_4^{\text{init}}, s_{\beta_0}(\cdot;\boldsymbol{\eta})\right\rangle \frac{s_{\beta_0}(\cdot;\boldsymbol{\eta})}{\|s_{\beta_0}(\cdot;\boldsymbol{\eta})\|} - \left\langle h_4^{\text{init}}, s_{\beta_1}(\cdot;\boldsymbol{\eta})\right\rangle \frac{s_{\beta_1}(\cdot;\boldsymbol{\eta})}{\|s_{\beta_1}(\cdot;\boldsymbol{\eta})\|} - \left\langle h_4^{\text{init}}, s_{\phi}(\cdot;\boldsymbol{\eta})\right\rangle \frac{s_{\phi}(\cdot;\boldsymbol{\eta})}{\|s_{\phi}(\cdot;\boldsymbol{\eta})\|}$$
$$- \left\langle h_4^{\text{init}}, h_0\right\rangle \frac{h_0}{\|h_0\|} - \left\langle h_4^{\text{init}}, h_3\right\rangle \frac{h_3}{\|h_3\|}.$$

2.1.2 Modified Gram-Schmidt

The procedure described above is mathematically correct, but numerically imprecise, as rounding errors may accumulate. Instead the following sequence is calculated:

$$h_{3a}(y) = h_3^{init} - \left\langle h_3^{\text{init}}, s_{\beta_0}(\cdot;\boldsymbol{\eta})\right\rangle \frac{s_{\beta_0}(\cdot;\boldsymbol{\eta})}{\|s_{\beta_0}(\cdot;\boldsymbol{\eta})\|}$$

$$h_{3b}(y) = h_{3a}(y) - \langle h_{3a}, s_{\beta_1}(\cdot;\boldsymbol{\eta})\rangle \frac{s_{\beta_1}(\cdot;\boldsymbol{\eta})}{\|s_{\beta_1}(\cdot;\boldsymbol{\eta})\|}$$

$$h_{3c}(y) = h_{3b}(y) - \langle h_{3b}, s_{\phi}(\cdot;\boldsymbol{\eta})\rangle \frac{s_{\phi}(\cdot;\boldsymbol{\eta})}{\|s_{\phi}(\cdot;\boldsymbol{\eta})\|}$$

$$h_{3d}(y) = h_{3c}(y) - \langle h_{3c}, h_0\rangle h_0$$

This orthogonalizes with respect to errors in the previous orthogonalizations, a procedure called modified Gram-Schmidt (MGS) [6]. A similar procedure is applied for finding h_4.

It is important to note that since the score function for the dispersion $s_\phi(\cdot;\boldsymbol{\eta})$ is not polynomial, neither are the $h_j(y)$ functions anymore after the orthogonalization. The first two steps of the orthogonalization are still with respect to polynomial score functions $s_{\beta_0}(\cdot;\boldsymbol{\eta})$ and $s_{\beta_1}(\cdot;\boldsymbol{\eta})$, and the inner products can be found analytically using the moment-generating function. The orthogonalization with respect to the score function of the dispersion $s_\phi(\cdot;\boldsymbol{\eta})$ and between the functions h_3 and h_4 will have to occur numerically. This means that the sum defining the inner product as in (4) is truncated after a large quantile, e.g. 1-10^{-6}.

2.1.3 Visual representation of the basis functions

h_3 and h_4 are basis functions that have been optimized to capture deviations of the observed densities from the hypothesized negative binomial density f. As explained before, they are no longer polynomial, and it may be informative to explore their shape. Examples of h_3 and h_4 are plotted in Figure 1 for a negative binomial distribution with mean $\mu_i = 6.677$ and dispersion $\phi = 2$. Observations much larger than the mean indicate excessive skewness and kurtosis, as could have been expected. For small values the interpretation is

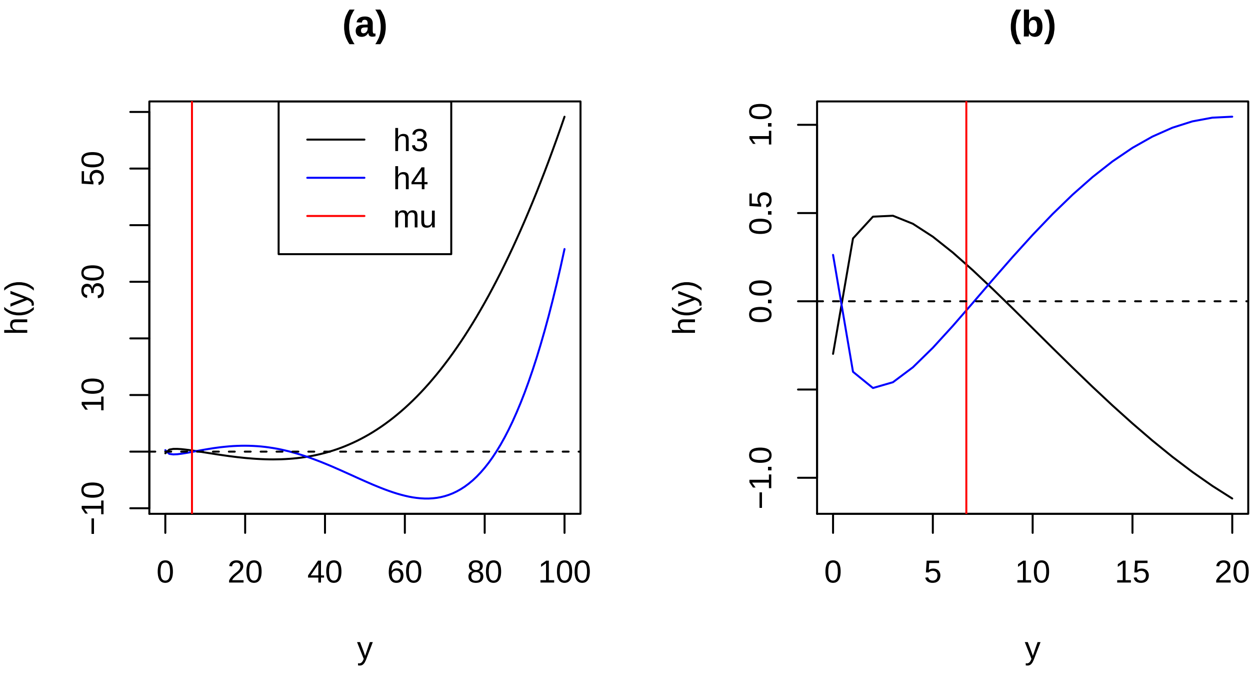

Figure 1: Function values of the two basis functions (y-axis) for different outcome values (x-axis). The same basis functions are shown on a large scale (a) and a small scale (b). The discrete basis functions are represented by straight lines between their function values. Vertical red line indicates the mean.

less straightforward. Evidently, these are only the basis functions for one particular combination of mean and dispersion parameters, but the similar trends are observed for other parameter values.

2.2 Smooth test statistic

2.2.1 Score statistics

The smooth test statistic is a quadratic form in the score statistics for θ_3 and θ_4 in (1). Let $L(\boldsymbol{\eta}, \boldsymbol{\theta})$ denote the likelihood function for a sample of n observations with density function (1). The score statistic for θ_j is given by

$$S_j(\boldsymbol{\eta}) = \frac{\partial}{\partial \theta_j} \log L(\boldsymbol{\eta}, \boldsymbol{\theta}) = \sum_{i=1}^{n} h_j(y_i; \boldsymbol{\eta}, \boldsymbol{x}_i).$$

Let

$$\boldsymbol{V}(\boldsymbol{\eta}) = \frac{1}{\sqrt{n}} \begin{pmatrix} S_3(\boldsymbol{\eta}) \\ S_4(\boldsymbol{\eta}) \end{pmatrix}.$$

The components of $\boldsymbol{V}$ are referred to as the components of the smooth test. When the unknown nuisance parameter $\boldsymbol{\eta}$ is replaced with its maximum likelihood estimate (MLE) under the null hypothesis, say $\hat{\boldsymbol{\eta}}$, the

variance of $\boldsymbol{V}(\hat{\boldsymbol{\eta}})$ (again under the null hypothesis) is given by

$$\mathrm{Var}_0\left\{\boldsymbol{V}(\hat{\boldsymbol{\eta}})\right\} = \boldsymbol{I}_{\theta\theta} - \boldsymbol{I}_{\theta\eta}\boldsymbol{I}_{\eta\eta}^{-1}\boldsymbol{I}_{\eta\theta},$$

in which $\boldsymbol{I}_{\theta\theta}$, $\boldsymbol{I}_{\theta\eta} = \boldsymbol{I}_{\eta\theta}^{t}$ and $\boldsymbol{I}_{\eta\eta}$ are elements of the information matrix

$$\boldsymbol{I} = \begin{pmatrix} \boldsymbol{I}_{\eta\eta} & \boldsymbol{I}_{\eta\theta} \\ \boldsymbol{I}_{\theta\eta} & \boldsymbol{I}_{\theta\theta} \end{pmatrix} = -\mathrm{E}_0\left\{\frac{\partial^2}{\partial(\boldsymbol{\eta},\boldsymbol{\theta})\partial(\boldsymbol{\eta},\boldsymbol{\theta})^t}\log L(\boldsymbol{\eta},\boldsymbol{\theta})\right\}.$$

The elements of this information matrix are given in the next section.

2.2.2 Information matrix

- The matrix $\boldsymbol{I}_{\theta\theta} = n\boldsymbol{I}_n$ (i.e. n times the identity matrix). This is a consequence of the orthonormality of the basis functions h_3 and h_4.

$$I_{\theta\theta} = \begin{bmatrix} n & 0 \\ 0 & n \end{bmatrix}$$

- The matrix $\boldsymbol{I}_{\eta\eta}^{-1}$ is by construction the variance-covariance matrix of the estimator of the parameter $\boldsymbol{\eta}$ of the negative binomial regression [7].

$$\boldsymbol{I}_{\eta\eta} = \begin{bmatrix} \sum_{i=1}^{n}\mu_i\frac{1+y_i\phi}{(1+\mu_i\phi)^2} & \sum_{i=1}^{n}\mu_i x_i\frac{1+y_i\phi}{(1+\mu_i\phi)^2} & 0 \\ \sum_{i=1}^{n}\mu_i x_i\frac{1+y_i\phi}{(1+\mu_i\phi)^2} & \sum_{i=1}^{n}\mu_i x_i^2\frac{1+y_i\phi}{(1+\mu_i\phi)^2} & 0 \\ 0 & 0 & \sum_{i=1}^{n}\Psi_3(1/\phi)-\Psi_3(y_i-1/\phi)-\phi+\frac{2}{\mu_i+1/\phi}-\frac{y_i+1/\phi}{(\mu_i+1/\phi)^2} \end{bmatrix}$$

  with Ψ_3 the trigamma function, the second derivative of the gamma function.

- Because of the imposed orthogonality of the basis functions and the score functions, all elements of the matrix $\boldsymbol{I}_{\theta\eta}$ are zero.

This means that simply

$$\mathrm{Var}_0\left\{\boldsymbol{V}(\hat{\boldsymbol{\eta}})\right\} = \boldsymbol{I}_{\theta\theta}.$$

2.2.3 Smooth test statistic

The smooth test statistic is then given by

$$T = \boldsymbol{V}(\hat{\boldsymbol{\eta}})^t \boldsymbol{I}_{\theta\theta}^{-1} \boldsymbol{V}(\hat{\boldsymbol{\eta}}).$$

References

[1] Neyman J. 'Smooth' test for goodness of fit. Skand Aktuarietidskr. 1937;20:150 – 199.

[2] Rayner JCW, Best DJ. Smooth Tests of Goodness of Fit: An Overview. International Statistical Review / Revue Internationale de Statistique. 1990;58(1):9–17.

[3] Rayner JCW, Thas O, Best DJ. Smooth Tests of Goodness of Fit: Using R. Wiley series in probability and statistics. Wiley; 2009. Available from: https://books.google.be/books?id=bDUEaflBSZ4C.

[4] Rippon P. Application of smooth tests of goodness of fit to generalized linear models; 2013. Available from: https://pdfs.semanticscholar.org/9683/bd5f6057d9f3bbf1b1f41ac8928dc7303911.pdf.

[5] Thas O. Comparing Distributions. Springer Series in Statistics. Springer New York; 2010. Available from: https://books.google.be/books?id=aQR_AAAAQBAJ.

[6] Rice JR. Experiments on Gram-Schmidt Orthogonalization. Mathematics of Computation. 1966;20(94):325 – 328.

[7] Lawless JF. Negative binomial and mixed Poisson regression. Canadian Journal of Statistics. 1987;15(3):209 – 225. doi:10.2307/3314912.
