# Supplementary material for: Sequence count data are poorly fit by the negative binomial distribution
Source: PLoS One. 2020 Apr 30;15(4):e0224909. doi: 10.1371/journal.pone.0224909 (PMC7192467; doi:10.1371/journal.pone.0224909)
Supplement: S2 Appendix — In this appendix more details are given about parametric bootstrap procedure for p-value calculations. (PDF) [file pone.0224909.s004.pdf]

S2 Appendix: The parametric bootstrap procedure

The parametric bootstrap procedure for goodness of fit hypothesis testing in the NB-model with B bootstrap runs, proceeds as follows (e.g. $B = 500$):

1. Using the n observed sample observations $(y_1, \ldots, y_n)$, estimate the β regression parameters and the overdispersion parameter ϕ in the NB-model with mean model $\mu(\boldsymbol{x}; \boldsymbol{\beta}) = \exp(\beta_0 + \boldsymbol{\beta}^t \boldsymbol{x})$. The parameter estimates are denoted by $\hat{\boldsymbol{\beta}}$ and $\hat{\phi}$.
2. Initiate the bootstrap run counter $b = 1$.
3. For each of the observed $\boldsymbol{x}_i$, $i = 1, \ldots, n$, sample at random (pseudorandom generator),

$$Y_i^* \mid \boldsymbol{x}_i \sim \text{NB}(\mu(\boldsymbol{x}_i; \hat{\boldsymbol{\beta}}), \hat{\phi}).$$

   This results in a bootstrap sample $(y_{b1}^*, \ldots, y_{bn}^*)$.

4. Using the bootstrap sample from the previous step, estimate the β and ϕ parameters according to the same NB regression model from step 1. The resulting estimates are denoted as $\hat{\boldsymbol{\beta}}_b^*$ and $\hat{\phi}_b^*$. Based on these estimates and the bootstrap sample, the goodness of fit test statistic (denoted by T_b) is computed.
5. If $b < B$, increase the bootstrap run counter with one, i.e. $b \leftarrow b + 1$ and go back to step 3. Otherwise go to the next step.
6. The set $\{T_1, \ldots, T_B\}$ represents the bootstrap sample of test statistics. Its empirical distribution is now used as the null distribution for p-value calculations.
