# Supplementary material for: Sequence count data are poorly fit by the negative binomial distribution
Source: PLoS One. 2020 Apr 30;15(4):e0224909. doi: 10.1371/journal.pone.0224909 (PMC7192467; doi:10.1371/journal.pone.0224909)
Supplement: S3 Appendix — In this appendix results of a simulation study are presented, aimed at investigating the sampling distribution of the MLE of the overdispersion parameter for a range of overdispersion values. (PDF) [file pone.0224909.s005.pdf]

S3 Appendix: Simulation study for studying the sampling distribution of the MLE of the overdispersion parameter

We investigate the sampling distribution of the MLE of the overdispersion parameter for various values of ϕ. The simulation settings were the same as in the simulation study presented in the paper, except that no differential abundance/expression was introduced and the sample size was set to $n = 100$. For the dispersion parameter ϕ values $\{0.001, 0.01, 0.1, 1, 5, 10, 50, 2, 500, 1000\}$ were used, and for each value 1000 samples were generated under the NB distribution. Next, the dispersion parameter was estimated through maximum likelihood.

As can be seen from Figures **??**-**??**, the MLE of the dispersion parameter is unbiased in the range 0.1-100, but only for ϕ close to 1 the estimator follows a normal distribution. For values ≥ 5 or ≤ 0.1, the distribution of the estimator is right skewed. Note that on the log-scale a strong bimodality of the distribution of the estimator is visible.

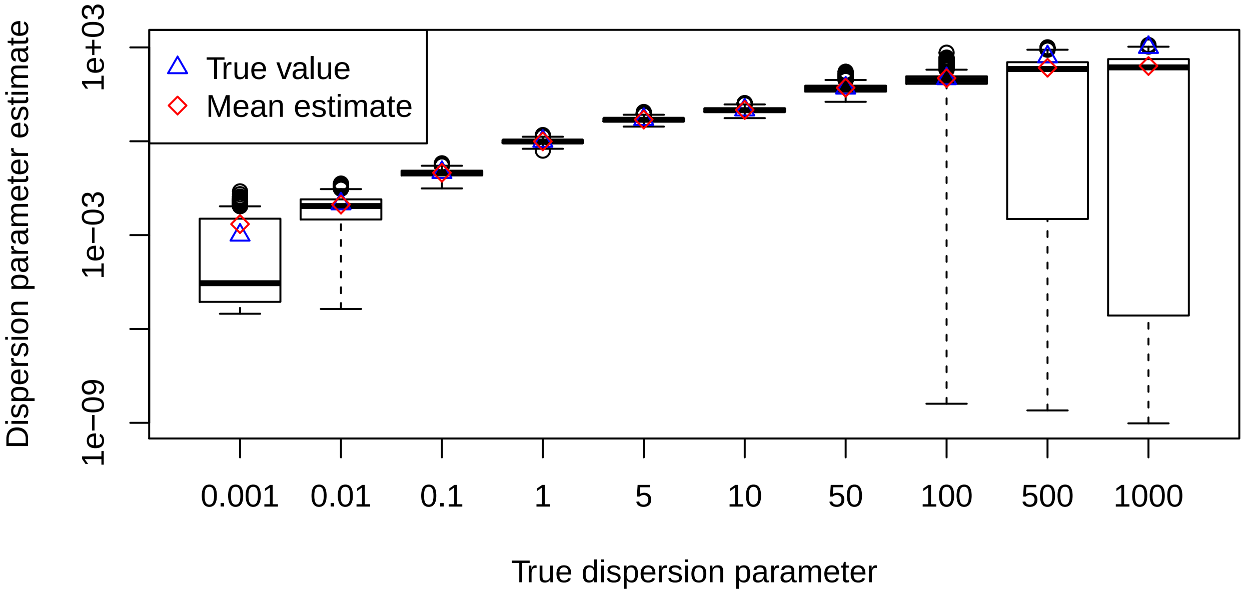

Figure 1: Boxplots of estimated dispersion parameters. The blue triangles and values on the x-axis represent the true values, while the red diamonds are the average estimates.

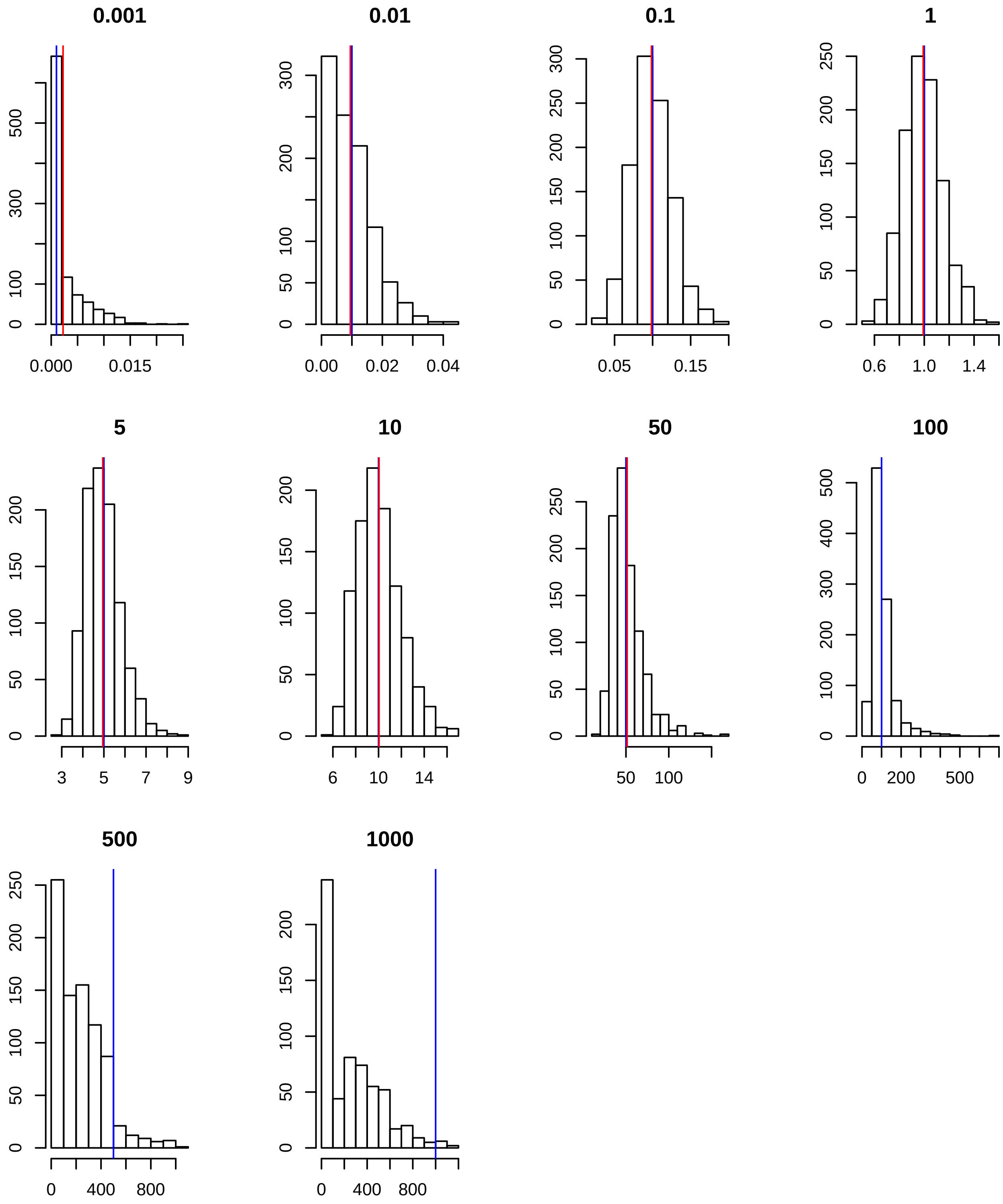

Figure 2: Histograms of dispersion estimates. The blue line is the true value, the red line the average estimate. The title of each graphs also shows the true dispersion.

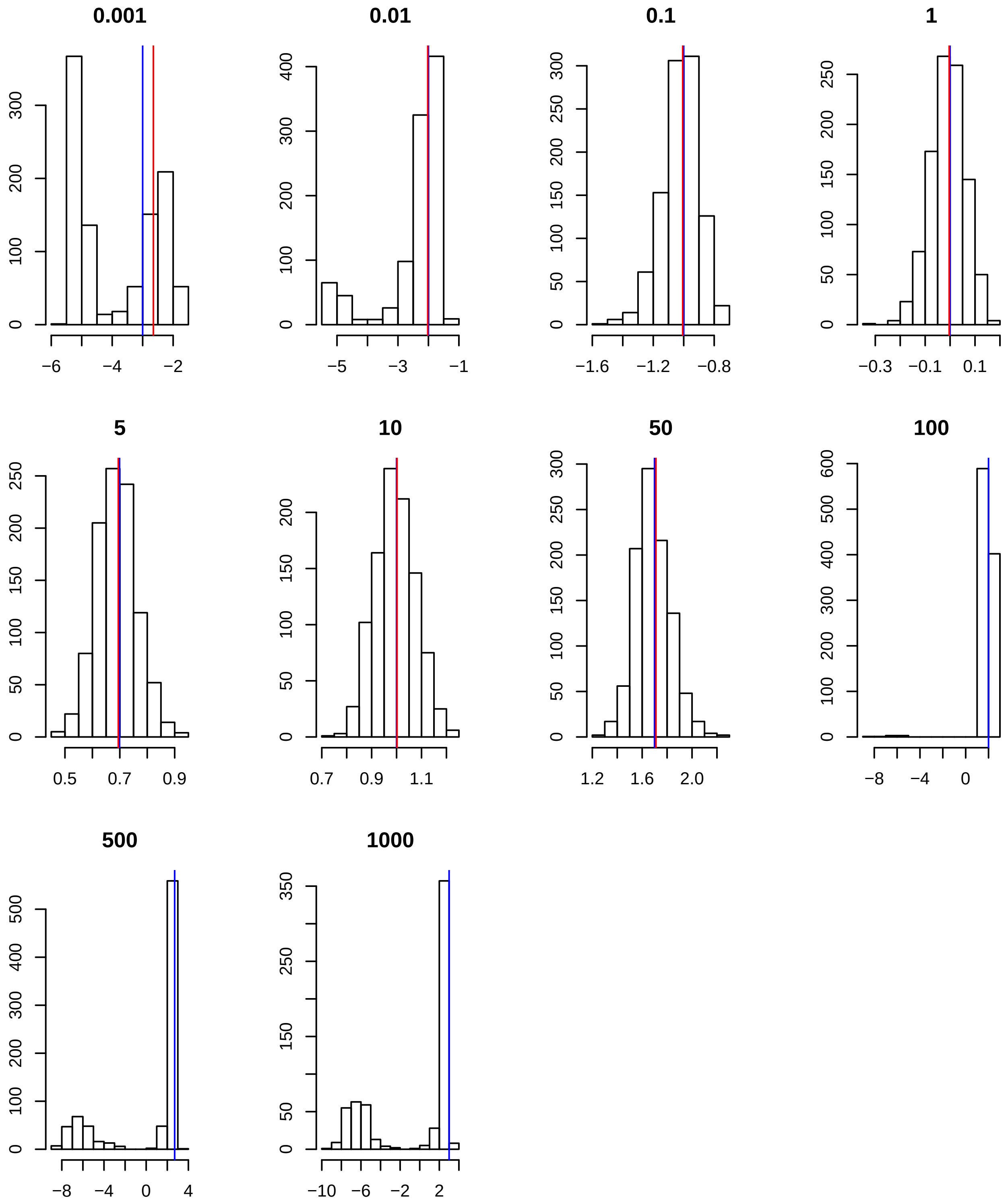

Figure 3: Histograms of log10-dispersion estimates. The blue line is the true value, the red line the average estimate. The title of each graphs also shows the true dispersion.
