# Supplementary material for: Sequence count data are poorly fit by the negative binomial distribution
Source: PLoS One. 2020 Apr 30;15(4):e0224909. doi: 10.1371/journal.pone.0224909 (PMC7192467; doi:10.1371/journal.pone.0224909)
Supplement: S4 Appendix — In this appendix details are provided about the datasets used in the paper. (PDF) [file pone.0224909.s006.pdf]

# S4 Appendix: Datasets

We investigated the following datasets for lack of fit to the NB distribution:

- **American Gut Project (AGP)** [1]: A large dataset on the stool microbiome of healthy human volunteers.
- **Human Microbiome Project (HMP)** [2]: A large dataset of healthy human volunteers, with microbiome samples from many body sites. Subsets of this dataset from the following body regions will be used:
  - Skin
  - Vagina
  - Oral cavity
- **Colorectal cancer (Zeller)** [3]: The colorectal microbiome of healthy and colorectal cancer patients. Sequence counts are rounded prior to analysis.
- **Armpit** [4]: Swabs from armpit microbiomes of human volunteers.
- **Crohn's disease** [5]: Gut microbiome of healthy and Crohn's diseased patients.
- **Colorectal cancer (Kostic)** [6]: The colorectal microbiome of healthy and colorectal cancer patients.
- **Squirrels** [7]: Seasonal changes of the gut microbiome of squirrels.
- **Humanized mice** [8]: Gut microbiomes of gnotobiotic mice that were inoculated with human feces, and then put on different diets.
- **Cooling water** [9]: The bacterial communities of a cooling water circuit of a nuclear test plant.
- **Lakes** [10]: Microbiome samples from lakes Muskegon and Michigan throughout different seasons.
- **Keyboard** [11]: Swabs of the microbiome found on keys of a keyboard and on human fingertips.
- **Neuroblastoma (cell line)** [12]: RNA-Seq dataset of neuroblastoma cell line data, treated by nutlin or ethanol.
- **Human brain** [13]: RNA-Seq dataset of human gene expression in hypothalamus and hippocampus from the GTEx project.
- **Neuroblastoma (human)** [14]: RNA-Seq dataset of neuroblastoma. Two groups are formed by tumors with and without amplification of the MYCN gene.

On each of these datasets, negative binomial regression models were fit with one sample-specific variable as regressors. These variables were chosen for their biological interest, e.g. as potential candidate for differential expression/abundance testing. The sample-specific variables used, as well as a categorization of these datasets in terms of their origin and sequencing technology are shown in the following table:

| Dataset | Grouping variable | Origin | Sequencing technology |
|---|---|---|---|
| AGP | IBD status | Human | Illumina MiSeq |
| HMP skin | Subregion | Human | 454 pyrosequencing |
| HMP vagina | Subregion | Human | 454 pyrosequencing |
| HMP oral cavity | Subregion | Human | 454 pyrosequencing |
| Colorectal cancer (Zeller) | Cancer diagnosis | Human | Illumina MiSeq |
| Armpit | Gender | Human | Illumina MiSeq |
| Crohn's disease | IBD status | Human | Illumina MiSeq |
| Colorectal cancer (Kostic) | Cancer diagnosis | Human | 454 pyrosequencing |
| Squirrels | Hibernation state | Mammal | 454 pyrosequencing |
| Humanized Mice | Diet | Mammal | 454 pyrosequencing |
| Keyboard | Sample location | Inert surface | 454 pyrosequencing |
| Cooling water | Reactor phase | Freshwater | Illumina MiSeq |
| Lakes | Lake | Freshwater | Illumina MiSeq |
| Neuroblastoma (cell line) | Ethanol/nutlin treatment | RNA-Seq | Illumina MiSeq |
| Human brain | Brain region | RNA-Seq | Illumina MiSeq |
| Neuroblastoma (human) | MYCN amplification | RNA-Seq | Illumina MiSeq |

Table 1: Overview of the datasets under study and their characteristics

Other choices of grouping variables are possible in the datasets under study. Also, including more variables into the NB regression model may improve the fit to the NB distribution. Still for simplicity and comparability of the results on the different datasets we stick to this simple design.

## References


[1] AmericanGut org. The American gut project. 2015;.

[2] Peterson J, Garges S, Giovanni M, McInnes P, Wang L, Schloss JA, et al. The NIH Human Microbiome Project. Genome Res. 2009;19(12):2317–2323. doi:10.1101/gr.096651.109.

[3] Zeller G, Tap J, Voigt AY, Sunagawa S, Kultima JR, Costea PI, et al. Potential of fecal microbiota for early-stage detection of colorectal cancer. Mol Syst Biol. 2014;10(766). doi:10.15252/msb.20145645.

[4] Callewaert C, Lambert J, Van de Wiele T. Towards a bacterial treatment for armpit malodour. Experimental Dermatology. 2017;26(5):388 – 391. doi:10.1111/exd.13259.

[5] Vandeputte D, Kathagen G, Hoe KD, Vieira-Silva S, Valles-Colomer M, Sabino J, et al. Quantitative microbiome profiling links gut community variation to microbial load. Nature. 2017;551:507 – EP.

[6] Kostic AD, Gevers D, Pedamallu CS, Michaud M, Duke F, Earl AM, et al. Genomic analysis identifies association of Fusobacterium with colorectal carcinoma. Genome Res. 2012;22(2):292 – 298. doi:10.1101/gr.126573.111.

[7] Carey HV, Walters WA, Knight R. Seasonal restructuring of the ground squirrel gut microbiota over the annual hibernation cycle. Am J Physiol Regul Integr Comp Physiol. 2013;304(1):33 – 42. doi:10.1152/ajpregu.00387.2012.

[8] Turnbaugh PJ, Hamady M, Yatsunenko T, Cantarel BL, Duncan A, Ley RE, et al. A core gut microbiome in obese and lean twins. Nature. 2009;457(7228):480 – 484. doi:10.1038/nature07540.

[9] Props R, Kerckhof FM, Rubbens P, De Vrieze J, Hernandez Sanabria E, Waegeman W, et al. Absolute quantification of microbial taxon abundances. The ISME Journal. 2016;11:584 – 587.

[10] Props R, Schmidt ML, Heyse J, Vanderploeg HA, Boon N, Denef VJ. Flow cytometric monitoring of bacterioplankton phenotypic diversity predicts high population-specific feeding rates by invasive dreissenid mussels. Environ Microbiol. 2018;20(2):521 – 534. doi:10.1111/1462-2920.13953.

[11] Fierer N, Lauber CL, Zhou N, McDonald D, Costello EK, Knight R. Forensic identification using skin bacterial communities. Proc Natl Acad Sci USA. 2010;107(14):6477 – 6481. doi:10.1073/pnas.1000162107.

[12] Assefa AT, Paepe KD, Everaert C, Mestdagh P, Thas O, Vandesompele J. Differential gene expression analysis tools exhibit substandard performance for long non-coding RNA-sequencing data. Genome Biol. 2018;19:96. doi:10.1186/s13059-018-1466-5.

[13] Lonsdale J, Thomas J, Salvatore M, Phillips R, Lo E, Shad S, et al. The Genotype-Tissue Expression (GTEx) project. Nature Genetics. 2013;45:580 – EP.

[14] Zhang W, Yu Y, Hertwig F, Thierry-Mieg J, Zhang W, Thierry-Mieg D, et al. Comparison of RNA-seq and microarray-based models for clinical endpoint prediction. Genome Biol. 2015;16(1):133. doi:10.1186/s13059-015-0694-1.
