# Supplementary material for: Sequence count data are poorly fit by the negative binomial distribution
Source: PLoS One. 2020 Apr 30;15(4):e0224909. doi: 10.1371/journal.pone.0224909 (PMC7192467; doi:10.1371/journal.pone.0224909)
Supplement: S5 Appendix — In this appendix the relationship between the fit to the NB distribution and some data-related features are graphically investigated. (PDF) [file pone.0224909.s007.pdf]

S5 Appendix: Exploration of the lack of fit

Descriptive exploration of the type of lack of fit is performed as follows. Estimated dispersion parameters, mean relative abundances and proportion of zero counts are plotted as a function of raw p-values and feature wise deviances [1, 2]. Additionally, sample-wise deviances are plotted versus library sizes.

1 P-values

Estimated dispersions and mean relative abundances appear uncorrelated with the raw p-values (Figures 1-2). In the microbiome datasets, features with many zero abundances tend to have larger raw p-values (see Figure 3). Presumably, this is because for features with many zeroes, the power to reject a null hypothesis of the NB distribution is lower.

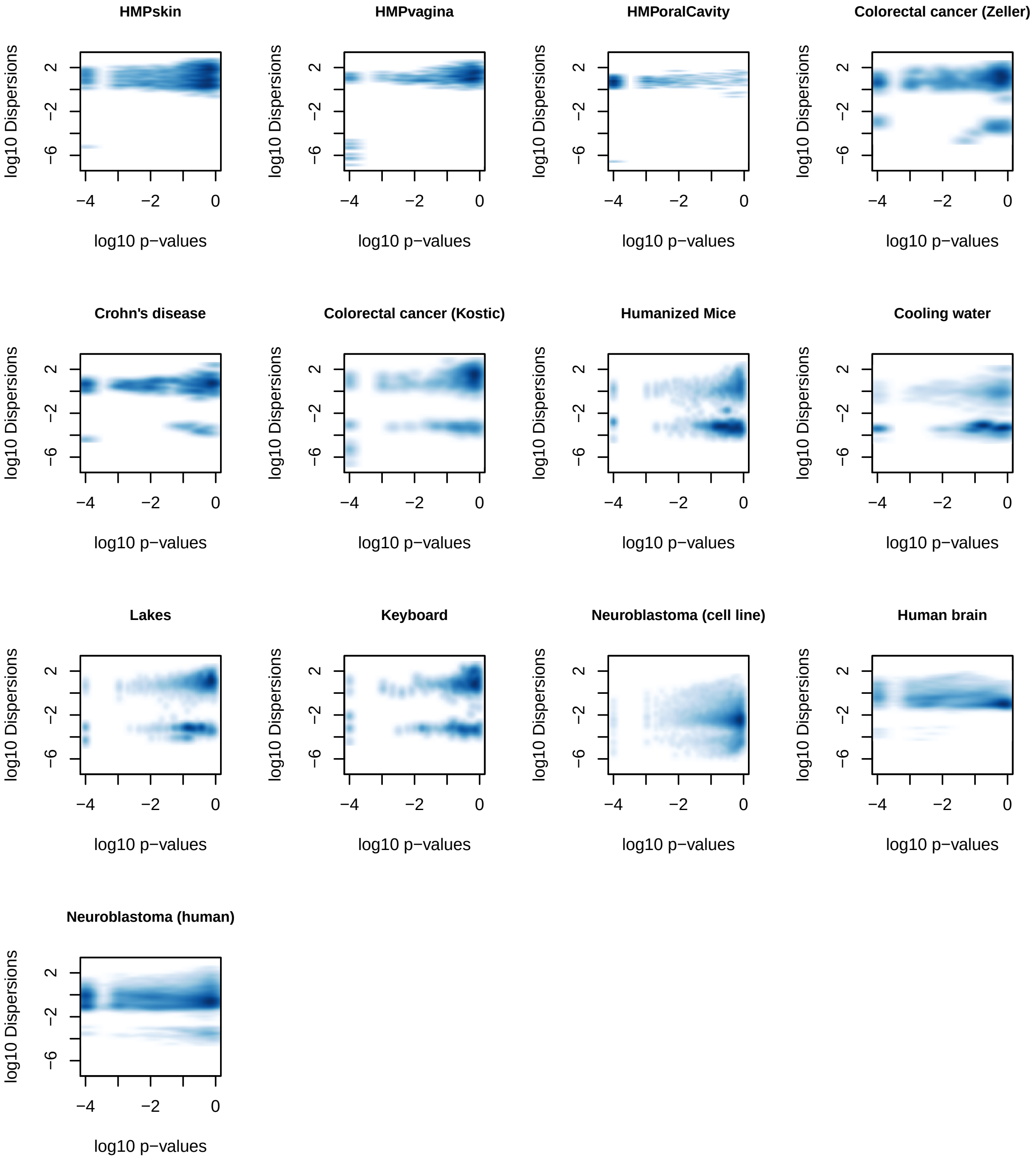

Figure 1: Density plots of raw p-values vs. estimated dispersions.

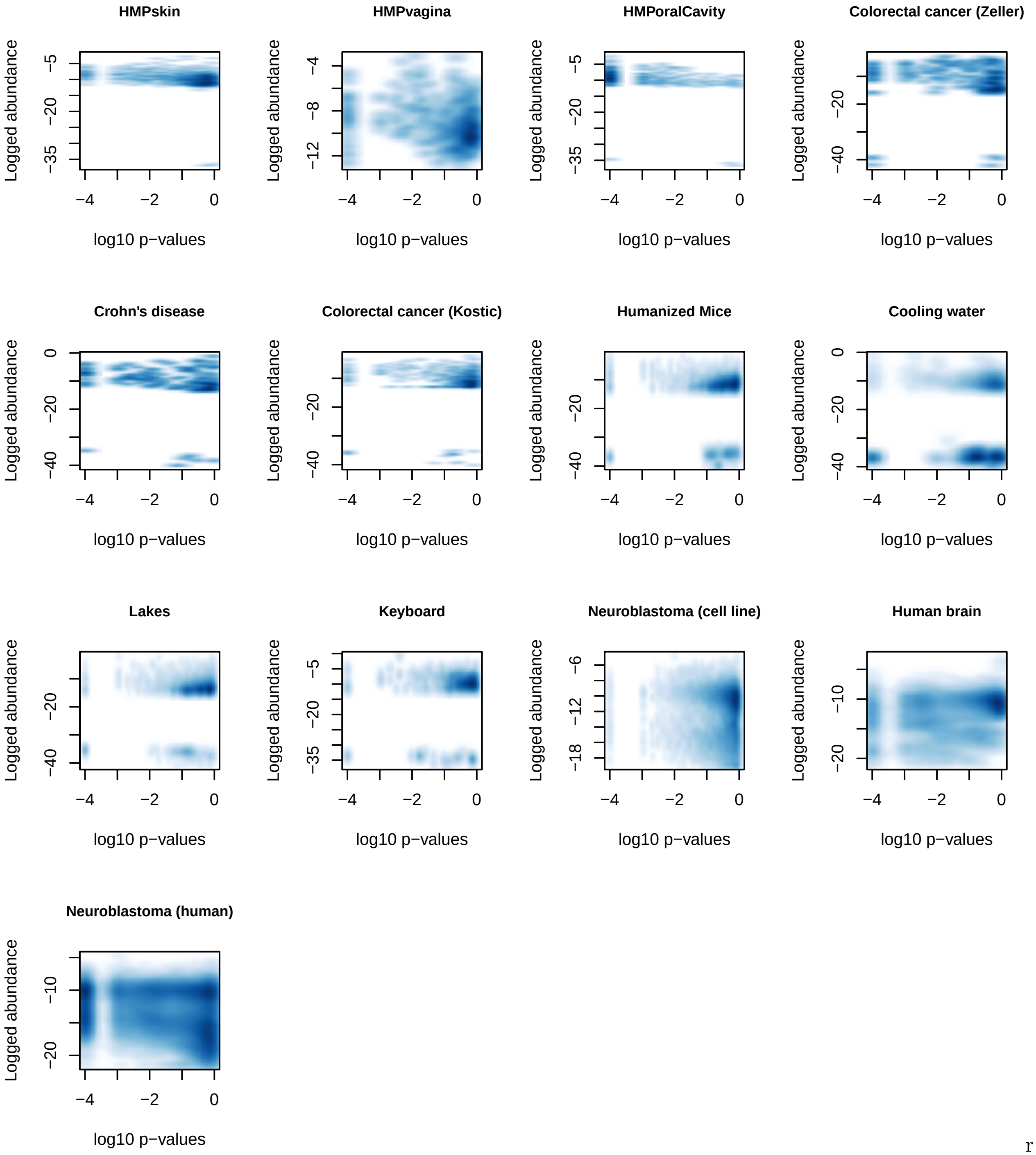

r

Figure 2: Density plots of raw p-values vs. logged estimated mean relative abundances.

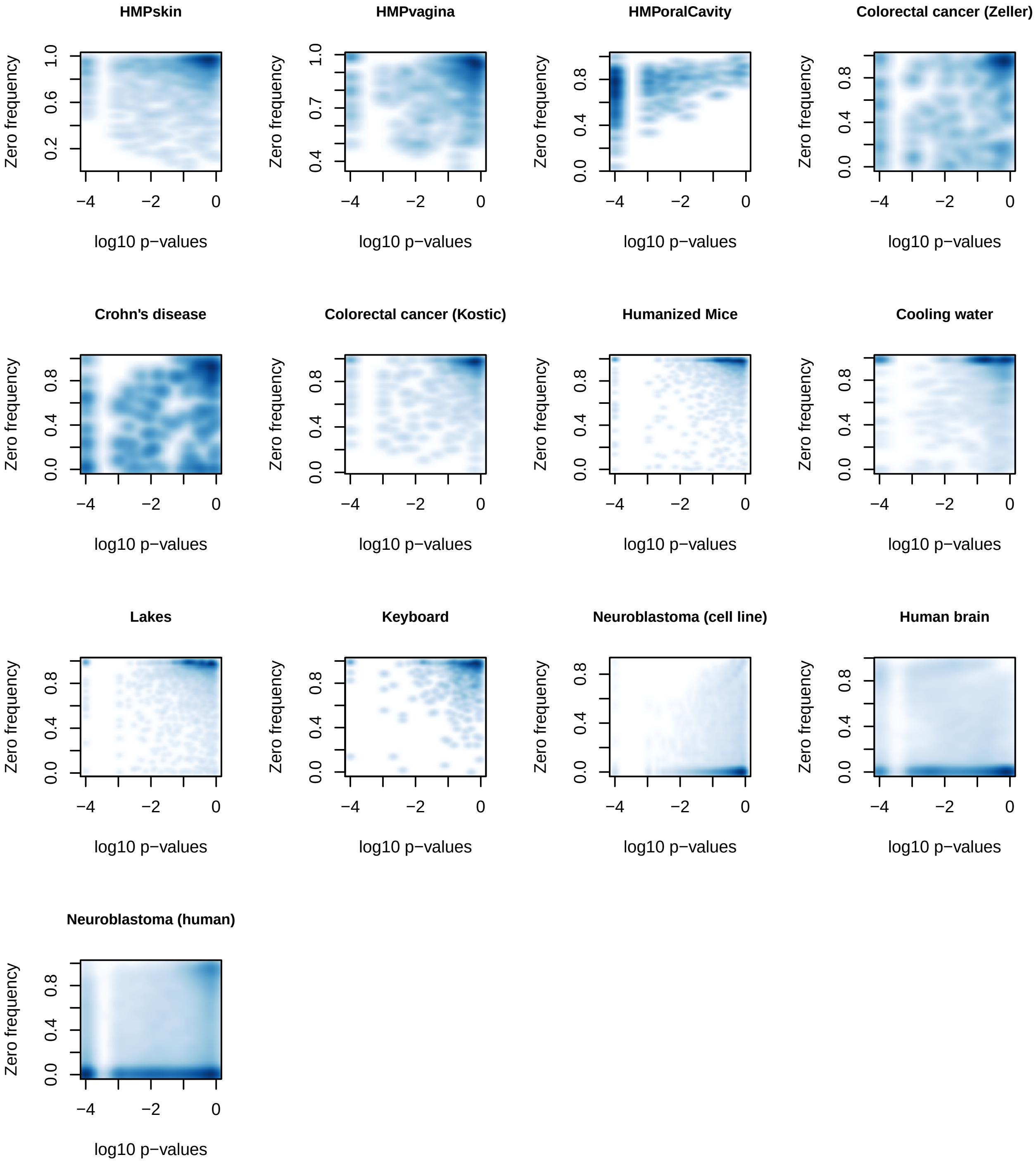

Figure 3: Density plots of raw p-values vs. zero frequencies.

2 Deviances

The sample-wise deviances are larger in samples with high library sizes (see Figure 4). Feature-wise deviances are larger in abundant features and in features with intermediate zero frequencies (see Figures 5 and 6)

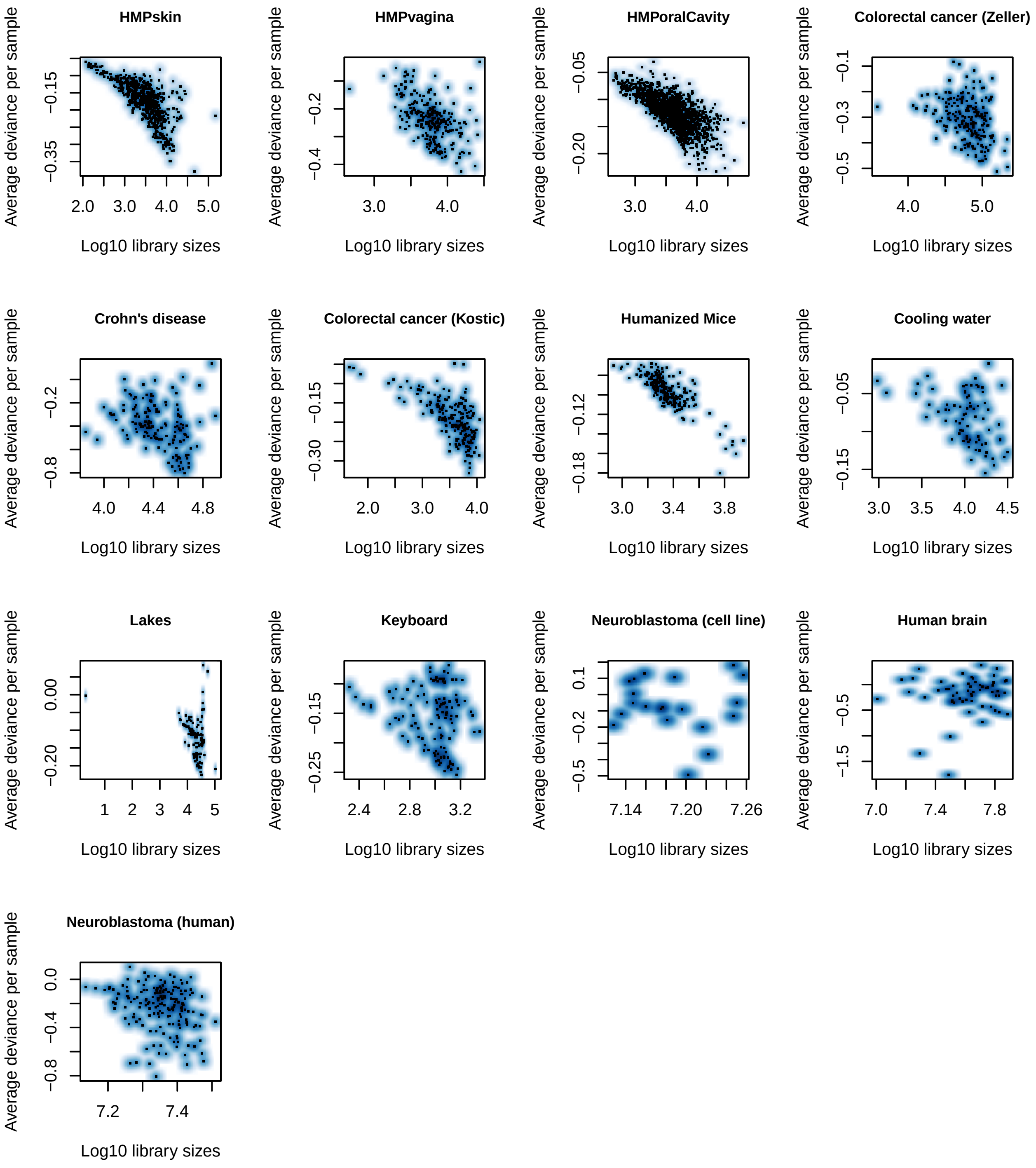

Figure 4: Density plots of average deviance per sample versus library sizes.

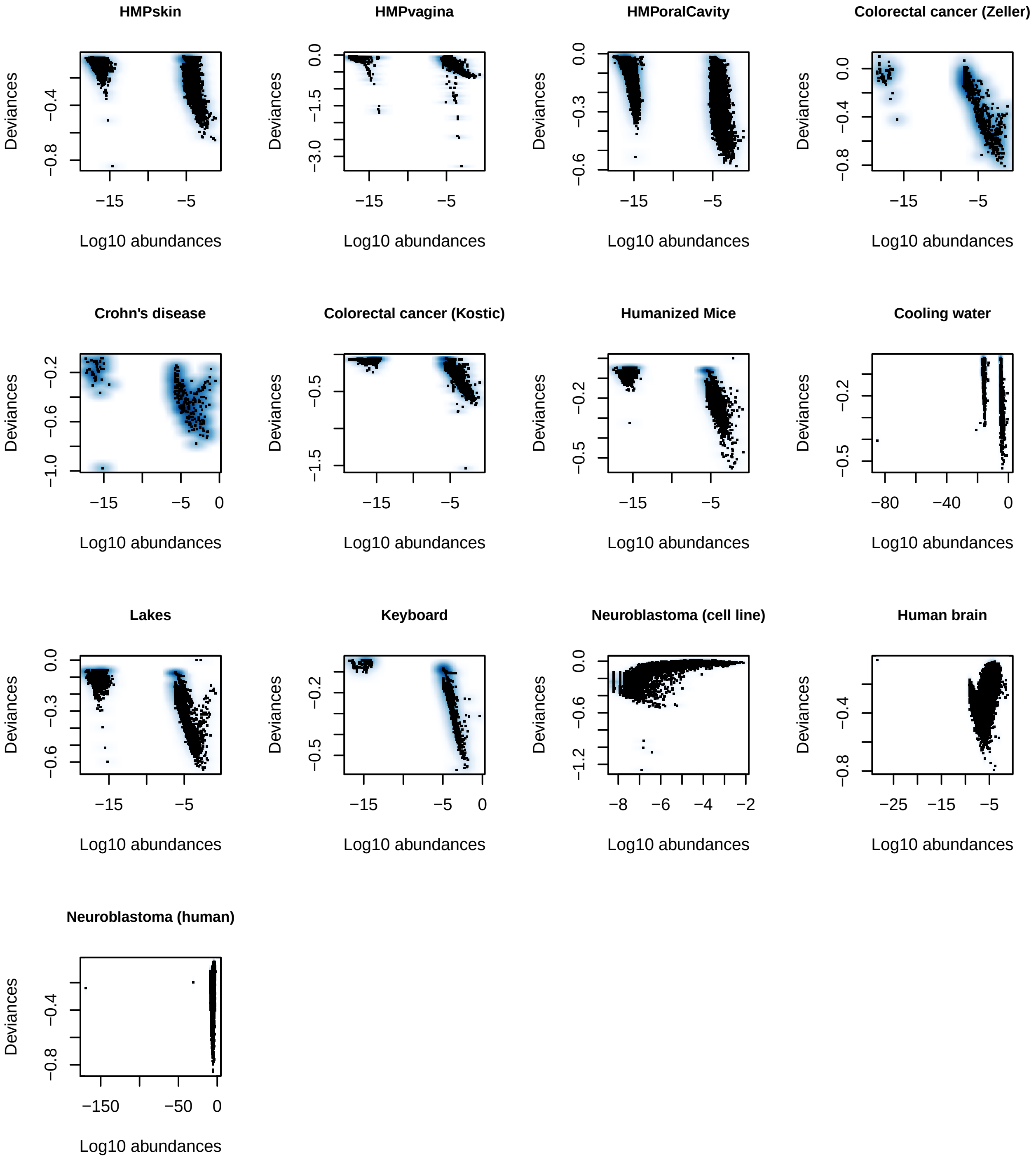

Figure 5: Density plots of average deviance per feature versus logged abundance.

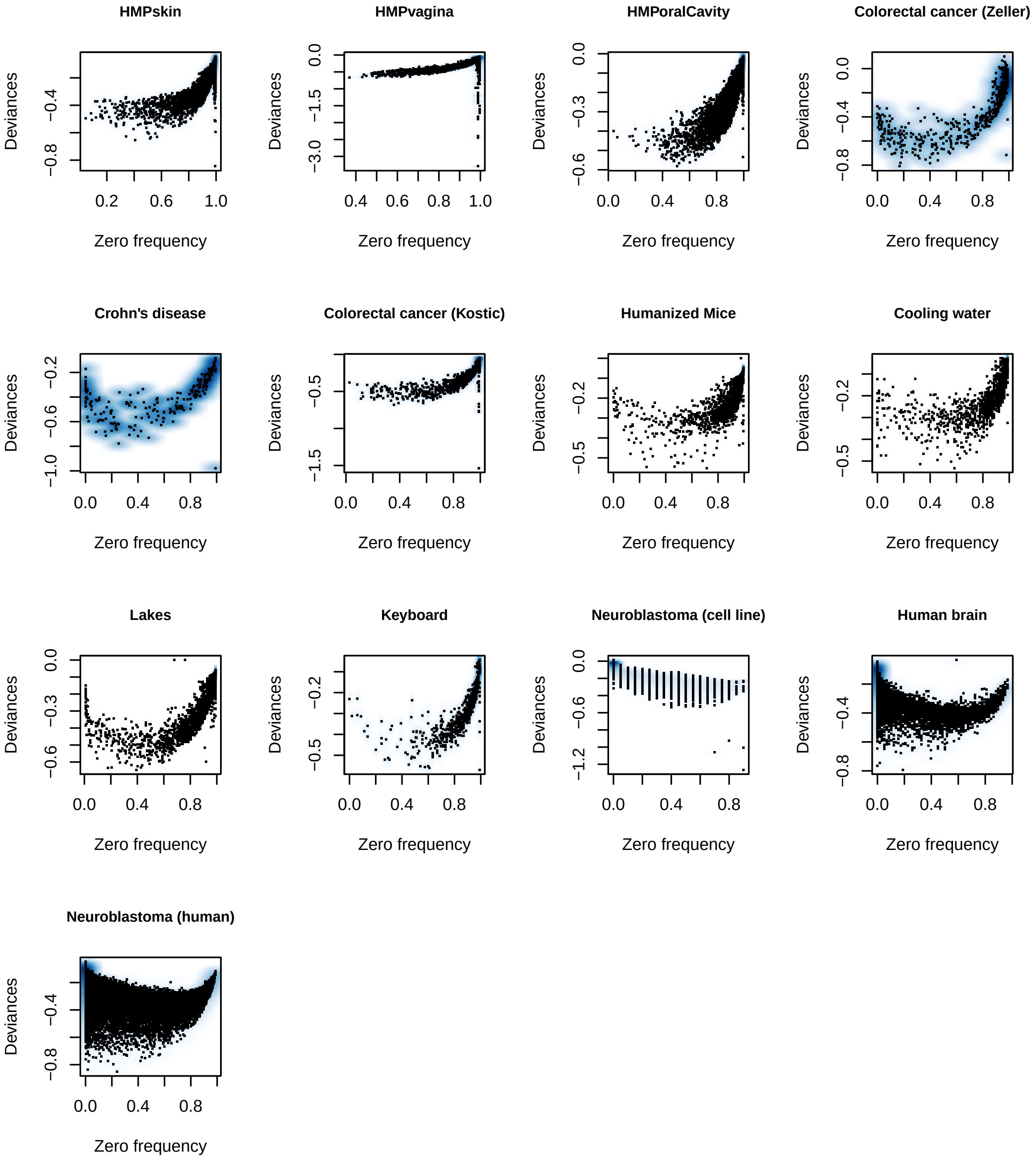

Figure 6: Density plots of average deviance per feature versus zero frequency.

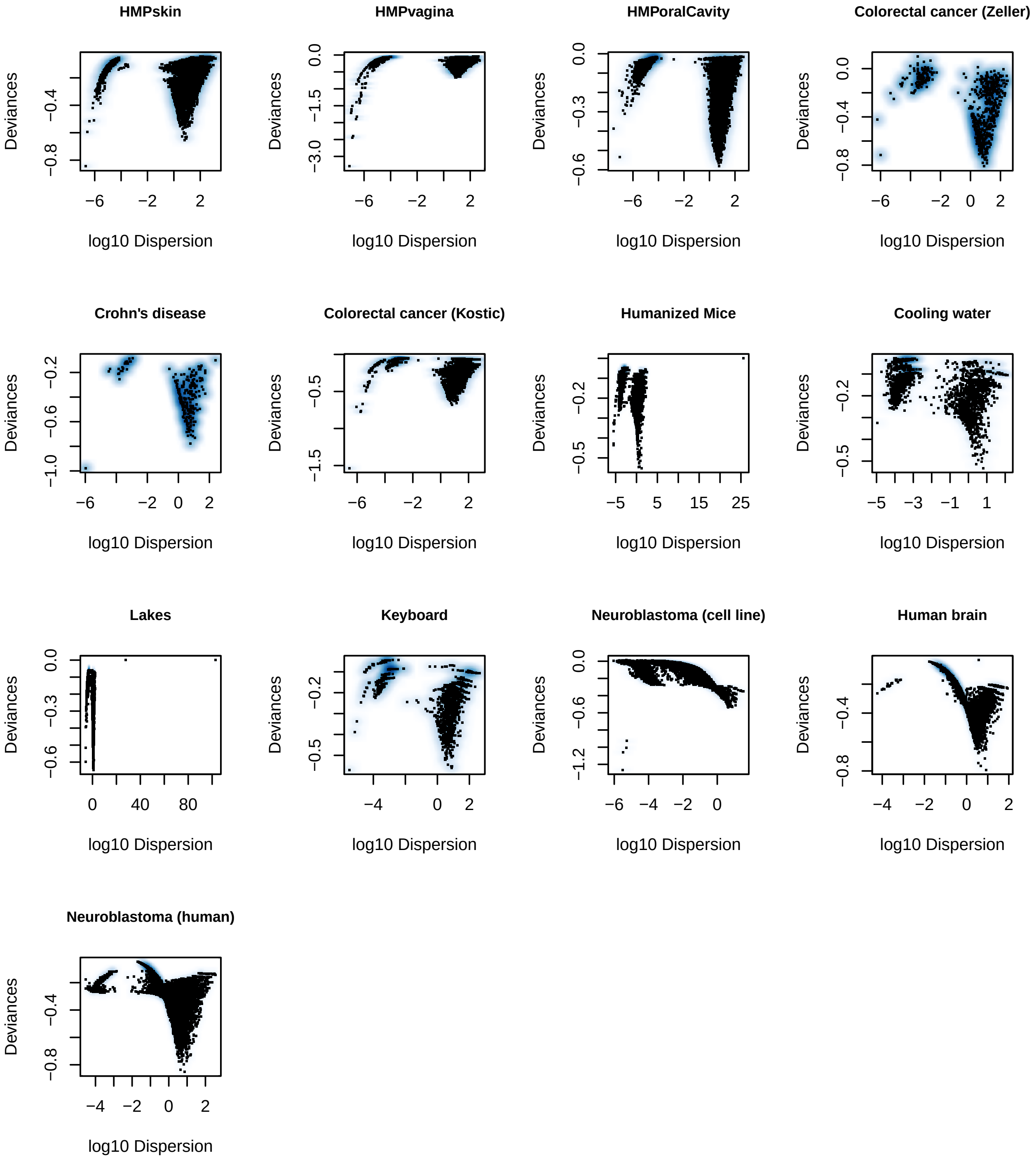

Figure 7: Density plots of average deviance per feature versus log10 dispersion.

References

[1] Zwilling ML. Negative Binomial Regression. The Mathematica Journal. 2013; p. 15 – 16. doi:10.3888/tmj.15-6.

[2] McCullagh P, Nelder JA. Generalized Linear Models, Second Edition. Chapman & Hall/CRC Monographs on Statistics & Applied Probability. Taylor & Francis; 1989.
