# Supplementary material for: Sequence count data are poorly fit by the negative binomial distribution
Source: PLoS One. 2020 Apr 30;15(4):e0224909. doi: 10.1371/journal.pone.0224909 (PMC7192467; doi:10.1371/journal.pone.0224909)
Supplement: S1 Table — Proportions of features without zero observations in all datasets under study. (PDF) [file pone.0224909.s009.pdf]

S1 Table. Zero observations. Proportions of features without zero observations in all datasets under study.

Dataset	Proportion of features without zeroes
American gut project	0.00
HMPskin	0.00
HMPvagina	0.00
HMPoralCavity	0.00
Colorectal cancer (Zeller)	0.00
Armpit	0.00
Crohn's disease	0.06
Colorectal cancer (Kostic)	0.00
Humanized Mice	0.00
Squirrels	0.00
Cooling water	0.00
Lakes	0.00
Keyboard	0.00
Neuroblastoma (cell line)	0.81
Human brain	0.76
Neuroblastoma (human)	0.54
